# Accelerated OH Oxidation of Methyl Chloride via Surface Accumulation at the Air–Water Interface

**DOI:** 10.1021/acsphyschemau.6c00076

**Published:** 2026-08-19

**Authors:** Yutong Yang, Chung Chi Chio, Ying-Lung Steve Tse

**Affiliations:** Department of Chemistry, 26451 The Chinese University of Hong Kong, Sha Tin, Hong Kong, China

**Keywords:** molecular dynamics, empirical valence bond, air−water interface, on-water reaction, reactivity at interfaces, solvation

## Abstract

Chemical reactions often proceed differently at the air–water interface than in bulk solution. In particular, “on-water” catalysis refers to cases where reactions appear to be accelerated at aqueous surfaces. A growing body of work shows that rate acceleration can arise from intertwined effects, including modified solvation and acid–base behavior, as well as enhanced reactant concentrations. Despite this progress, it remains difficult to quantify, for specific atmospheric reactions, how much interfacial rate enhancement arises from (i) intrinsic changes in the free energy barrier versus (ii) concentration-driven effects, because fully converged interfacial free energy profiles are costly to obtain with ab initio molecular simulations. Here, we address this challenge for the reaction of methyl chloride CH_3_Cl with hydroxyl radicals (OH), an important atmospheric sink of CH_3_Cl, by developing a reaction-specific empirical valence bond (EVB) model parametrized against high-level ab initio data. The EVB model, combined with a flexible polarizable water force field, enables efficient condensed-phase simulations and systematic comparison of gas phase, bulk aqueous, and interfacial reactivity. Our results show that the reaction free energy barrier is only weakly affected by proximity to the air–water interface, whereas thermodynamic enrichment of the surface-active reactants increases the effective interfacial rate by more than 2 orders of magnitude relative to the aqueous bulk and by over 6 orders of magnitude relative to the gas phase.

## Graphical abstract

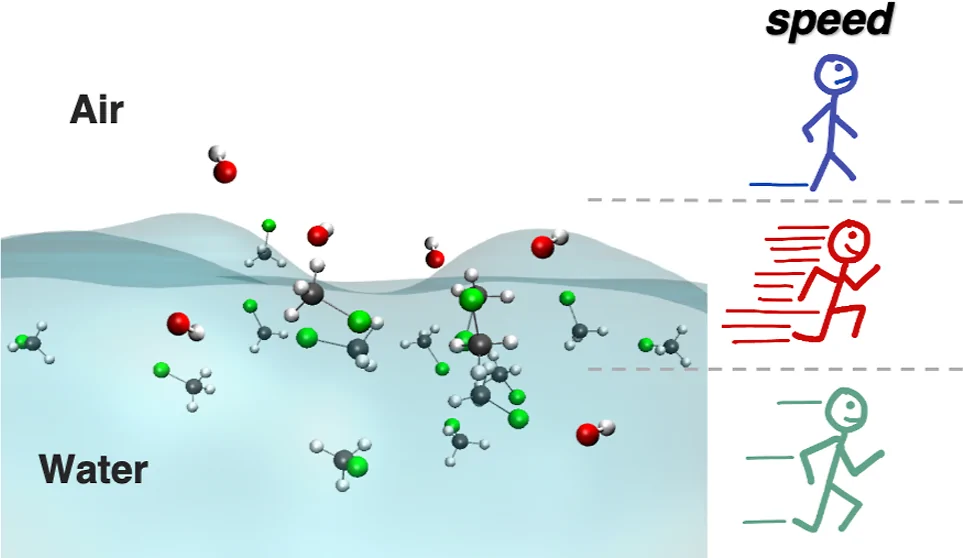

## 1 Introduction

When certain organic reactions are carried out by simply mixing the reactants with water and stirring vigorously, the resulting suspension of small droplets can display markedly enhanced reaction rates compared to reactions in a conventional organic solvent. Sharpless and co-workers^1^ systematically studied such organic reactions in aqueous suspensions and coined the term “on-water” for this type of reaction. While on-water catalysis is well established in organic chemistry, similar effects are also believed to play an important role in atmospheric chemistry but are much less understood, despite the air–water interface of cloud and aerosol droplets being ubiquitous.^2,3^ Consequently, understanding the chemistry at aqueous interfaces has drawn increasing attention and is essential for evaluating the atmospheric budgets of many trace gases.^4,5^

A growing body of experimental and theoretical work has demonstrated that the air–water interface and related aqueous interfaces can substantially concentrate organic and ionic species, leading to altered reactivity compared with bulk solution.^6–8^ Organic solutes frequently adsorb strongly at hydrophobic–water interfaces, resulting in interfacial concentrations that can exceed bulk concentrations by orders of magnitude. This interfacial enrichment has been identified as a straightforward and often dominant mechanism for rate enhancement in microdroplets and at aqueous surfaces.^6^ In atmospheric contexts, organic films and aqueous aerosols can similarly concentrate reactive species, modify their orientation and local environment, and thereby promote reactions that are inefficient or even disfavored in bulk solution.^7^ More broadly, recent reviews of molecular reactions at aqueous interfaces highlight that rate acceleration can arise from several intertwined effects, including modified solvation, distinct acid–base behavior, interfacial electric fields, and enhanced reactant concentrations at the interface.^8^

Despite these advances, for most specific atmospheric reactions it remains challenging to quantify the relative contributions of (i) intrinsic barrier changes and (ii) concentration-driven effects to interfacial rate enhancements. This difficulty stems in part from the high computational cost of obtaining fully converged free energy profiles at realistic aqueous interfaces using ab initio molecular dynamics (AIMD), and in part from the subtle interplay between solvation structure, local electric fields, and interfacial partitioning.^6,8^ In this work, we address this challenge for the reaction between methyl chloride (CH_3_Cl) and hydroxyl radicals (OH), an important atmospheric sink of CH_3_Cl, by developing a reactive empirical valence bond (EVB) model^9^ that enables systematic and quantitative comparison of bulk, interfacial, and gas-phase reactivity.

The depletion of CH_3_Cl, a notable atmospheric pollutant, has received increasing attention since it can significantly impact the stratospheric ozone depletion process by generating chlorine atoms through UV radiation-induced photolysis.^10^ To better understand the atmospheric chemistry of CH_3_Cl and assess the role of the air–water interface, we aim to model and analyze the hydrogen abstraction reaction between CH_3_Cl and the OH radical, producing CH_2_Cl radical and H_2_O

1 $${CH}_{3}Cl+OH\to {CH}_{2}Cl+H_{2}O$$

This is one of the main sinks of CH_3_Cl in the atmosphere. The gas phase kinetics of this reaction have been extensively studied. Experimental reaction rate constants have been reported by Demore et al.^11^ and Sander et al.,^12^ with both studies giving a rate constant of 3.6 × 10^–14^ cm^3^ molecule^–1^ s^–1^ at 298 K, although the corresponding Arrhenius parameters differ. Using this rate constant, Yoshida et al.^13^ performed a three-dimensional global modeling study and obtained CH_3_Cl global losses consistent with the values reported by Koppmann et al.^14^ and by Khalil and Rasmussen.^15^

Previous quantum mechanical (QM) studies have examined the CH_3_Cl + OH reaction in the gas phase^16–18^ and, more recently, at aqueous interfaces.^19^ In particular, Martins-Costa et al.^19^ used density-functional theory (DFT) to explore the reaction at the air–water interface and reported activation barriers that are broadly consistent with gas-phase values, while suggesting that interfacial OH radicals may play an important role. These pioneering calculations, however, necessarily relied on relatively small interfacial models and limited sampling. Here we complement and extend that work by constructing a reaction-specific EVB model trained against high-level TightPNO DLPNO–CCSD­(T)^20–24^ reference data and applying it to large droplets and slabs containing up to 2000 water molecules. This approach allows us to obtain statistically converged free energy profiles in bulk water and at the air–water interface, and to disentangle, within a single framework, the contributions of barrier modulation and interfacial partitioning to the overall rate enhancement.

In the following, we first describe the modeling framework and EVB methodology, including the simulation setups used for bulk water and interfacial systems. We then present gas-phase benchmarks against high-level QM calculations, followed by free energy profiles in bulk and interfacial environments obtained from enhanced-sampling simulations. Finally, we use the combination of reaction barriers and solvation free energies to quantify, for this specific reaction, the relative roles of intrinsic barrier changes and interfacial enrichment in controlling on-water reactivity.

## 2 Simulations Methods

Classical molecular dynamics (MD) simulations are a powerful tool for investigating physical and chemical processes at the molecular scale, but conventional fixed-topology force fields are not designed to describe bond rearrangements. Several families of reactive force fields have therefore been developed, including ReaxFF^25^ and related approaches, which treat bond formation and breaking within a unified parametrization. In parallel, ab initio MD (AIMD), typically paired with DFT, provides an explicit treatment of electronic degrees of freedom and can achieve good accuracy for chemical reactions with a suitable functional. However, AIMD remains computationally demanding, which severely limits the accessible length and time scales at realistic aqueous interfaces.

EVB models^9^ offer a complementary strategy in which separate classical force fields are constructed for the reactant and product states and then coupled through an off-diagonal term that hybridizes these states. In particular, multistate EVB (MS-EVB) models developed by Voth and co-workers have been extensively applied to hydrated excess protons at aqueous interfaces, providing molecular-level insights into the equilibrium and dynamical properties of the excess proton near hydrophobic surfaces and the air–water interface,^26–28^ demonstrating that EVB approaches can reliably and efficiently model proton transfer reactions in a variety of different aqueous environments. In this work, we take advantage of the same general framework by training a two-state EVB model on high-level QM reference data, thereby combining the efficiency of classical MD with the accuracy of TightPNO DLPNO–CCSD­(T)^20–24^ reference data for the CH_3_Cl + OH reaction.

### 2.1 MD with Classical Polarizable Force Fields

Electronic polarizability can be important for accurately modeling certain interfacial properties, particularly dielectric response and ion distributions.^29–31^ In classical force fields, several approaches have been developed to incorporate electronic polarization, including fluctuating charge,^32–35^ induced point dipole,^36–38^ and Drude oscillator.^30,31,39^ Among these, Drude oscillators are particularly efficient for constant *NVT* simulations because of the possible use of extended Lagrangian.^40^ Previously, our group developed the flexible and polarizable SWM4/Fw water model,^41^ which is based on the Drude oscillators. The flexibility of the water model is critical for modeling chemical reactions. In this work, we parametrized reactive force fields within the EVB framework. The existing SWM4/Fw was used for water, and the parameters for the other species and all cross-interactions were optimized as described below.

All the classical MD simulations were carried out with constant *NVT* dynamics in OpenMM 8.^42^ The temperatures of the nuclei and the Drude oscillators were controlled at 300 and 1 K, respectively, using dual Langevin thermostats.^43^ The time step was 0.5 fs.

When the system resides entirely in either the reactant or product basin, it is well described by traditional force fields. In this work, the functional form of the classical forces follows the general AMBER force field (GAFF)^44^ with additional Drude oscillator terms to represent electronic polarizability

2 $$\begin{array}{l} U_{sys}=\sum_{bonds}k_{b}{\left(r-r_{0}\right)}^{2}+\sum_{angles}k_{a}{\left(\theta -\theta_{0}\right)}^{2}\\ +\sum_{i<j}\left\{4\epsilon_{ij}\left[{\left(\frac{\sigma_{ij}}{r_{ij}}\right)}^{12}-{\left(\frac{\sigma_{ij}}{r_{ij}}\right)}^{6}\right]+k_{e}\frac{q_{i}q_{j}}{r_{ij}}\right\}\\ +\sum_{Drudes}k_{D}d^{2} \end{array}$$

where *k*_b_, *k*_a_, and *k*_D_ are the force constants; *r*_0_ and θ_0_ are the equilibrium bond lengths and angles; ϵ and σ are the LJ parameters; *k*_*e*_ is the Coulomb constant; *q* are the partial charges; and *d* are the distances between Drude oscillators and their parent atoms.

### 2.2 EVB Model

To model the chemical reaction, we employed a two-state EVB model that couples the classical force fields for the reactants and products. The state of the system is written as a linear combination of the two basis states (or called EVB states)

3 $$|\Psi \rangle =c_{1}|\phi_{1}\rangle +c_{2}|\phi_{2}\rangle$$

where |ϕ_1_⟩ and |ϕ_2_⟩ are the reactant and product states, respectively. Moreover

4 $$c_{1}^{2}+c_{2}^{2}=1$$

the lowest electronic eigenvalue, i.e., the potential energy on the Born–Oppenheimer potential energy surface (PES), is then given by the smaller root of the characteristic equation of the 2 × 2 EVB Hamiltonian matrix

5 $$E_{g}=\frac{1}{2}\left[\left(E_{11}+E_{22}\right)-\sqrt{{\left(E_{11}-E_{22}\right)}^{2}+4E_{12}^{2}}\right]$$

where $E_{ij}=\langle i|Ĥ|j\rangle$ and *Ĥ* is the Hamiltonian operator.

In EVB, the potential energies of |ϕ_1_⟩ and |ϕ_2_⟩, *E*_11_ and *E*_22_, are typically modeled by classical force fields (see eq 2). We define a collective variable (CV) δ as the reactant coordinate for H-abstraction, given by the difference between the distance *d*(C–H*) from the carbon atom to the transferring hydrogen H* and the distance *d*(O–H*) from the oxygen atom of the OH radical to H*

6 $$\delta =d\left(C-H^{*}\right)-d\left(O-H^{*}\right)$$

δ has been selected as the CV because it effectively monitors the breaking/formation of the O–H and C–H bonds. Additionally, it tracks the intrinsic reaction coordinate (IRC) closely in the reference data and allows for direct comparison with existing studies.^19^

Near the transition state (TS), the off-diagonal element *E*_12_ couples the two states and refines the PES in the crossing region. In this work, *E*_12_ is modeled as the product of a Gaussian function and a quadratic polynomial in δ

7 $$E_{12}=D\;\exp\left[-A{\left(\delta -d_{0}\right)}^{2}\right]\times \left(a\delta^{2}+b\delta +c\right)$$

where *D*, *A*, *a*, *b*, *c*, and *d*_0_ are adjustable parameters. In our calculations, we focused on the range −3.0 Å < δ < + 3.0 Å, which covers the transition from reactants to products through a TS located near δ = 0. Figure 1 illustrates the reaction coordinate and representative schematic configurations on the reactant and product sides.

**Figure 1:**
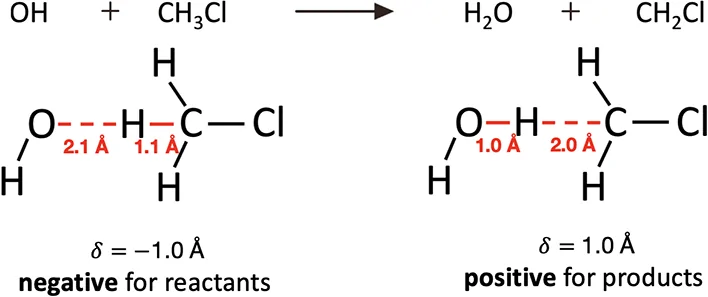
Reaction coordinate δ. See also eq 6 and its discussion in text.

### 2.3 EVB Model Parametrization

The reference data used for parametrizing the EVB model included.

1. Potential energies for 201 configurations of a cluster containing the reacting species and four additional water molecules (Figure 2). These structures were selected along the intrinsic reaction coordinate (IRC) using Gaussian 16 (ver. C.02).^45^ Geometry optimizations were performed with the M06–2X hybrid DFT functional^46^ including Grimme’s D3 dispersion correction^47^ and the ma-TZVP basis set.^48,49^ Single-point energies were then computed at a higher level of theory using TightPNO DLPNO–CCSD­(T)^20–24^ with the aug-cc-pVQZ basis set^50^ in ORCA.^51^ Benchmark results for several additional DFT functionals for this reaction are provided in the Supporting Information.
2. Binding energies of CH_3_Cl, CH_2_Cl radical, and OH radical with one to four water molecules, respectively. These calculations were performed at the same level of theory as the single-point energy calculations above. The counterpoise method was used to correct for basis set superposition error (BSSE).^52,53^

**Figure 2:**
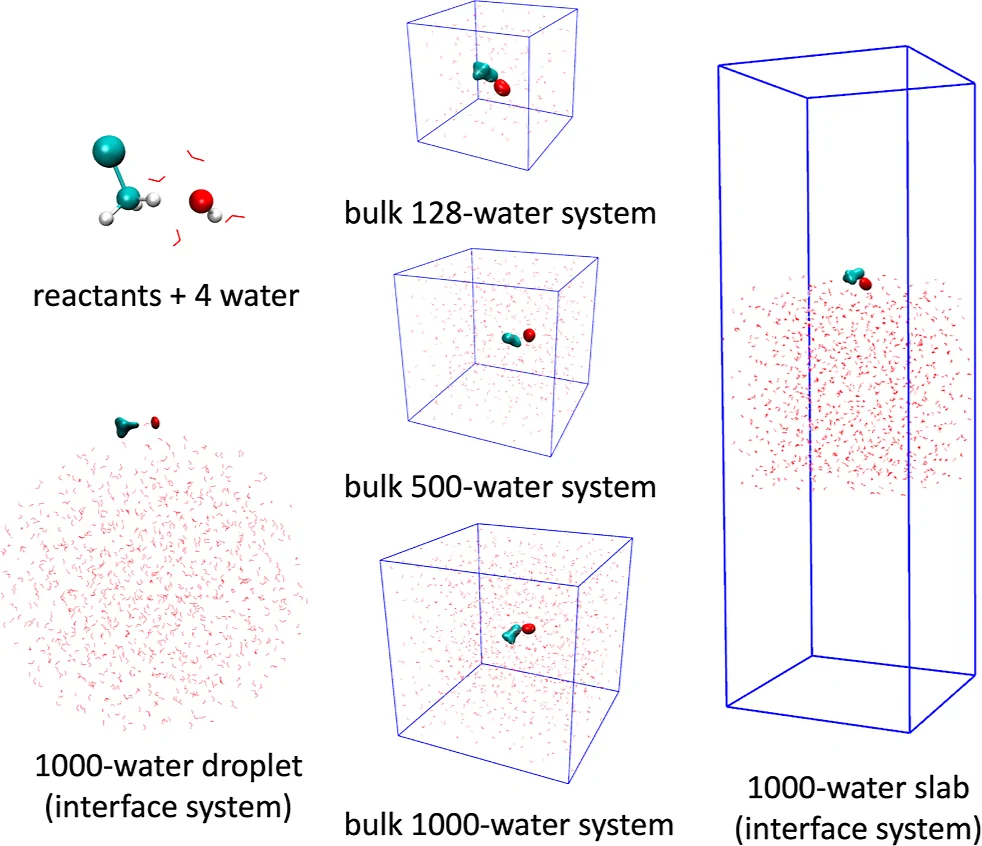
Snapshots of the systems studied in this work. For bulk and interface systems, CH_3_Cl and OH are shown as a cyan blob and a red blob, respectively.

The adjustable parameters of the EVB model were optimized by minimizing a loss function that combines the root-mean-square (rms) deviations of the potential energies (PE) and regularized binding energies (BE)

8 $$L=\langle \delta {PE\rangle }_{rms}+100\times \langle \delta {BE}_{reg}\rangle_{rms}$$

where

9 $$\langle \delta {PE\rangle }_{rms}={\left[\frac{1}{N_{PE}}\sum_{i=1}^{N_{PE}}{\left({PE}_{i}^{T}-{PE}_{i}^{M}\right)}^{2}\right]}^{1/2}$$

10 $$\langle \delta {BE}_{reg}\rangle_{rms}={\left[\frac{1}{N_{BE}}\sum_{i=1}^{N_{BE}}{\left(\frac{{BE}_{i}^{T}-{BE}_{i}^{M}}{{BE}_{i}^{T}}\right)}^{2}\right]}^{1/2}$$

Here, the superscripts T and M refer to the “Target” and “Model” values, respectively; *N*_PE_ and *N*_BE_ are the numbers of reference PE and BE data points; and the subscript “reg” refers to the regularized BE metric.

The optimization was performed using the covariance matrix adaptation evolution strategy (CMA-ES).^54^ Among the resulting candidate models, those with the lowest loss values were further examined, and a final model was selected based on its agreement with the reference data. The optimized parameters are listed in the Supporting Information. All results reported in this work were obtained with this optimized EVB model.

In addition to reproducing the PES and binding energies, we also tuned the model to better capture the interactions between water and OH radicals in the aqueous phase. Specifically, we adjusted the parameters to match the height of the first peak in the O–O* radial distribution function (RDF) *g*(*r*) to previously reported values from AIMD, where O* and O denote the oxygen atoms of the hydroxyl radical and water, respectively. With more details in a later section, the solvation structure of the OH radical in water compares well with the previously published AIMD results.

### 2.4 Simulation Systems

Using our EVB model, we studied the CH_3_Cl reaction in the gas phase, bulk water, and near the air–water interface, as illustrated in Figure 2. The simulation setups are summarized below.

1. **Gas phase:** The gas phase system included one CH_3_Cl, one OH radical, and four water molecules. This system is the same as the one we collected QM reference data to parametrize the EVB model. No periodic boundary conditions were used, and all the pairwise nonbonded interactions were calculated.
2. **Bulk water:** We simulated systems containing 128, 500, or 1000 water molecules in a periodic cubic box with side length of 15.7, 24.5, or 31.0 Å, respectively. These box lengths were chosen such that the liquid density in the *NVT* simulations matches the equilibrium density of the SWM4/Fw water model at 300 K that is about 1 g/cm^3^.^41^ The long-range Coulomb interactions were handled by the particle mesh Ewald (PME) method^55^ with a fractional force error tolerance of 1 × 10^–5^ and a real-space cutoff of 7.8, 12.2, or 15.5 Å for the systems including 128, 500, or 1000 water molecules, respectively. The Lennard-Jones (LJ) interactions used the same cutoff as the real-space cutoff used in PME, with an additional switching function applied at 1.0 Å to smoothly turn off the LJ at the cutoff.
3. **Air–water interface:** To study the reaction in the presence of the air–water interface, we simulated either a water droplet or a water slab subject to PBCs with 1000 water molecules. For the droplet, a roughly spherical cluster with a radius of ∼19.3 Å was placed in a nonperiodic simulation box, and all pairwise interactions were included without cutoffs. For the slab geometry, we used a periodic box of dimensions 31.0 × 31.0 × 100.0 Å^3^, with a water slab of thickness ∼31.0 Å along the *z*-axis. A cutoff of 15.5 Å was employed for both the LJ interactions and the real-space part of the PME calculations.

## 3 Results and Discussion

### 3.1 Potential Energy Surface (PES) in the Gas Phase

A reference gas-phase PES was calculated along the IRC and then mapped onto δ. The resulting PES as a function of δ is plotted in Figure 3, along with several representative structures. The TS exhibits a reactant-like configuration, leaning toward a state with a shorter C–H distance than the O–H distance (δ = *d*(C–H*) – *d*(O–H*) ≈ −0.22 Å). The forward and backward reaction barriers are approximately 30 and 107 kJ/mol, respectively. These findings are consistent with the findings of Martins-Costa et al.^19^ Beyond the energetics, we also examined the evolution of chemical bonding and noncovalent interactions along the reaction coordinate by an interaction region indicator (IRI) analysis (Figure S1 and Supporting Information Video S1).^56^

**Figure 3:**
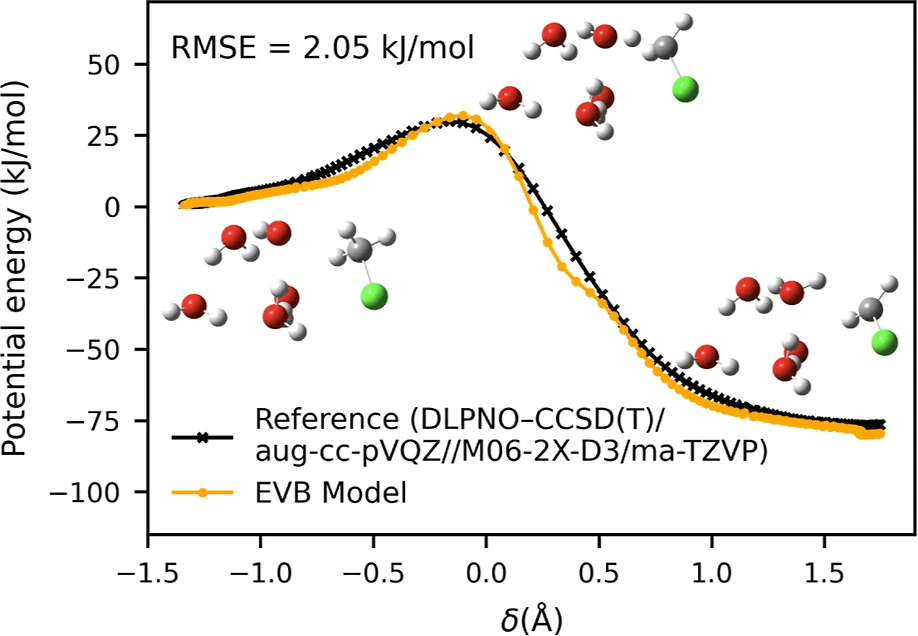
PES along IRC for the gas phase system containing the reactants and four water molecules. δ (see eq 6) is obtained by transforming the IRC coordinate. Snapshots for the reactants, TS, and products along the IRC are shown. Color code: (hydrogen, white), (oxygen, red), (carbon, gray), and (chlorine, green).

Our EVB model reproduces the reference PES accurately (Figure 3), with a root-mean-square error (RMSE) of ∼2.05 kJ/mol. The RMSE of regularized binding energies by the EVB model is ∼9.1% (see Supporting Information for details).

### 3.2 Solvation Structure of the OH Radical in Bulk Water

We first examined the solvation structure of the OH radical in bulk water by comparing the RDFs (i) $g_{O-O^{*}}\left(r\right)$ between water oxygen O and hydroxyl radical oxygen O*, and (ii) $g_{H-O^{*}}$ between water hydrogen H and O* using our EVB model and compared them with the previously published AIMD results by VandeVondele and Sprik,^57^ Genova et al.^58^ and Apostolidou^59^ as shown in Figure 4. The RDFs from EVB were calculated from an unbiased 50 ns trajectory for a periodic cubic box of 1000 water molecules and two OH radicals at 300 K. In general, the RDFs and the corresponding integrated coordination numbers by EVB are consistent with those of AIMD. In particular, for O–O*, all the curves exhibit a first peak at *r* ≈ 2.8–2.9 Å, and the peak height from our EVB model (about 2.3 for $g_{O-O^{*}}$ ) lies within the range reported in these AIMD studies.

**Figure 4:**
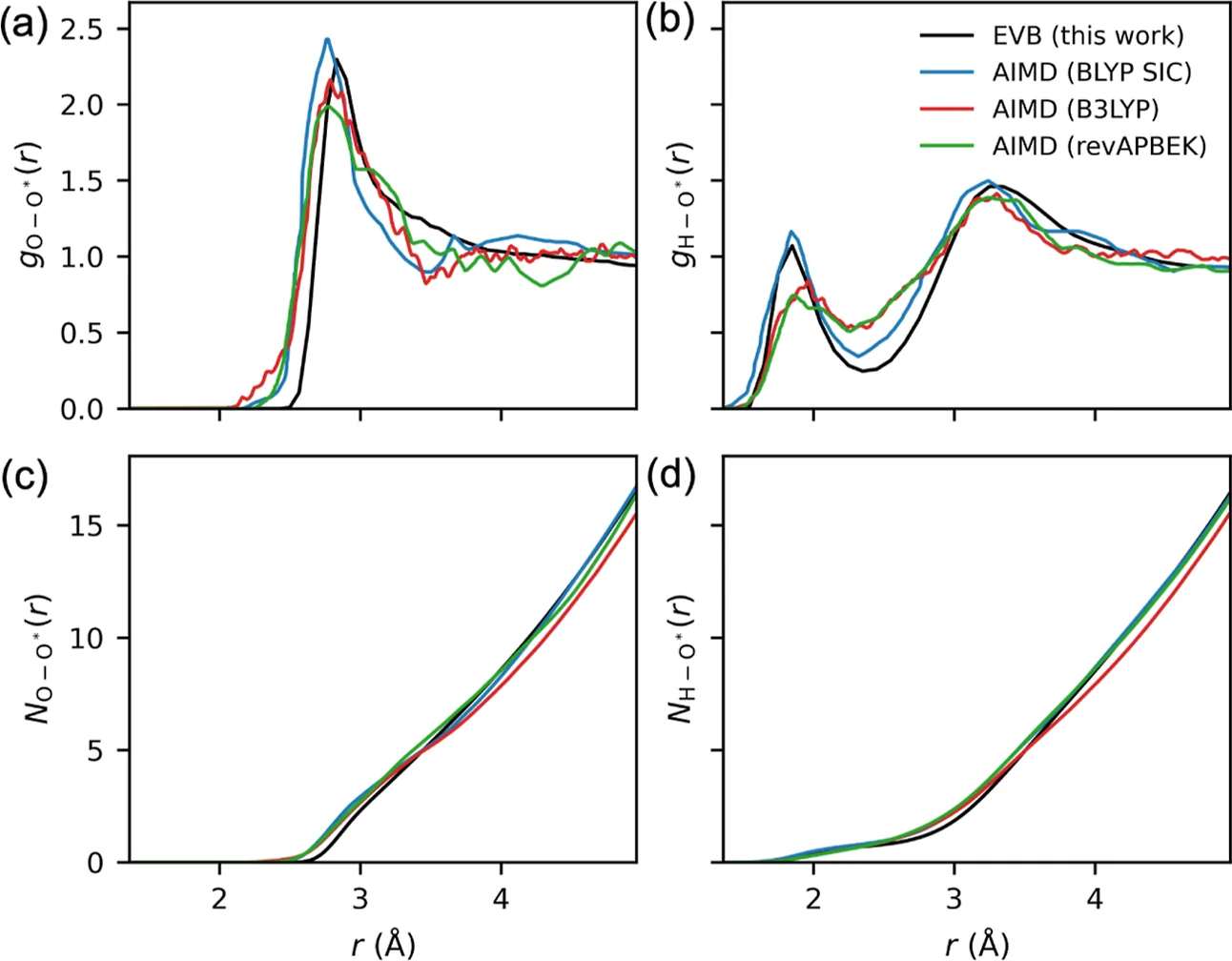
Solvation structure of the OH radical in a periodic cubic box of 1000 water molecules at 300 K. (a) O–O* RDF $g_{O-O^{*}}\left(r\right)$ ; (b) H–O* RDF $g_{H-O^{*}}\left(r\right)$ ; (c) integrated coordination number $N_{O-O^{*}}\left(r\right)$ ; (d) $N_{H-O^{*}}\left(r\right)$ . Here, O* denotes hydroxyl radical oxygen and H denotes water hydrogen. The EVB results are plotted in black, and the AIMD results for BLYP-SIC,^57^ B3LYP,^59^ and revAPBEK^58^ are plotted as blue, red, and green curves, respectively.

### 3.3 Free Energy Profiles in Gas and Condensed Phases

With the efficient EVB model, the free energy profiles of the reaction as a function of δ (eq 6) can be readily obtained in the gas and condensed phases (bulk water and air–water interface). We computed the free energy profiles by steered molecular dynamics (SMD).^60^ The work *W*(*z*) accumulated in each pull in these nonequilibrium simulations is related to the free energy change Δ*F* by the Jarzynski equality (JE)^61,62^

11 $$\Delta F\left(z\right)=-k_{B}T\;\ln\left\langle \exp\left(\frac{-W\left(z\right)}{k_{B}T}\right)\right\rangle$$

where ⟨·⟩ denotes an average over pulling trajectories that start from equilibrium configurations with the bias center fixed at the initial CV value. Specifically, SMD pulls were carried out by varying δ from −3.0 to 3.0 Å using a moving harmonic restraint with a pulling speed of 2 Å/ns and a spring constant of 1 × 10^4^ kJ/mol/Å^2^. This large force constant is justified by the large mean forces and the stiff-spring approximation.^60^ Initial configurations for each pull were extracted every 10 ps from an equilibrium trajectory, after 3 ns of equilibration with the bias fixed at the starting δ value. 150 SMD pulls were performed for each system.

For the gas-phase reaction with four explicit water molecules, the obtained free energy profile is shown as the black curve in Figure 5a; the forward reaction barrier is approximately 26 kJ/mol. This free energy barrier is slightly lower than the corresponding potential energy barrier of about 30 kJ/mol. Using the configurations of the TightPNO DLPNO–CCSD­(T) reference data, we evaluated the free energy along the IRC using Shermo.^63^ The resulting free energy profile in Figure S2 yields a forward barrier of about 26 kJ/mol, statistically the same value estimated by our EVB model. This excellent agreement further validates the EVB model.

**Figure 5:**
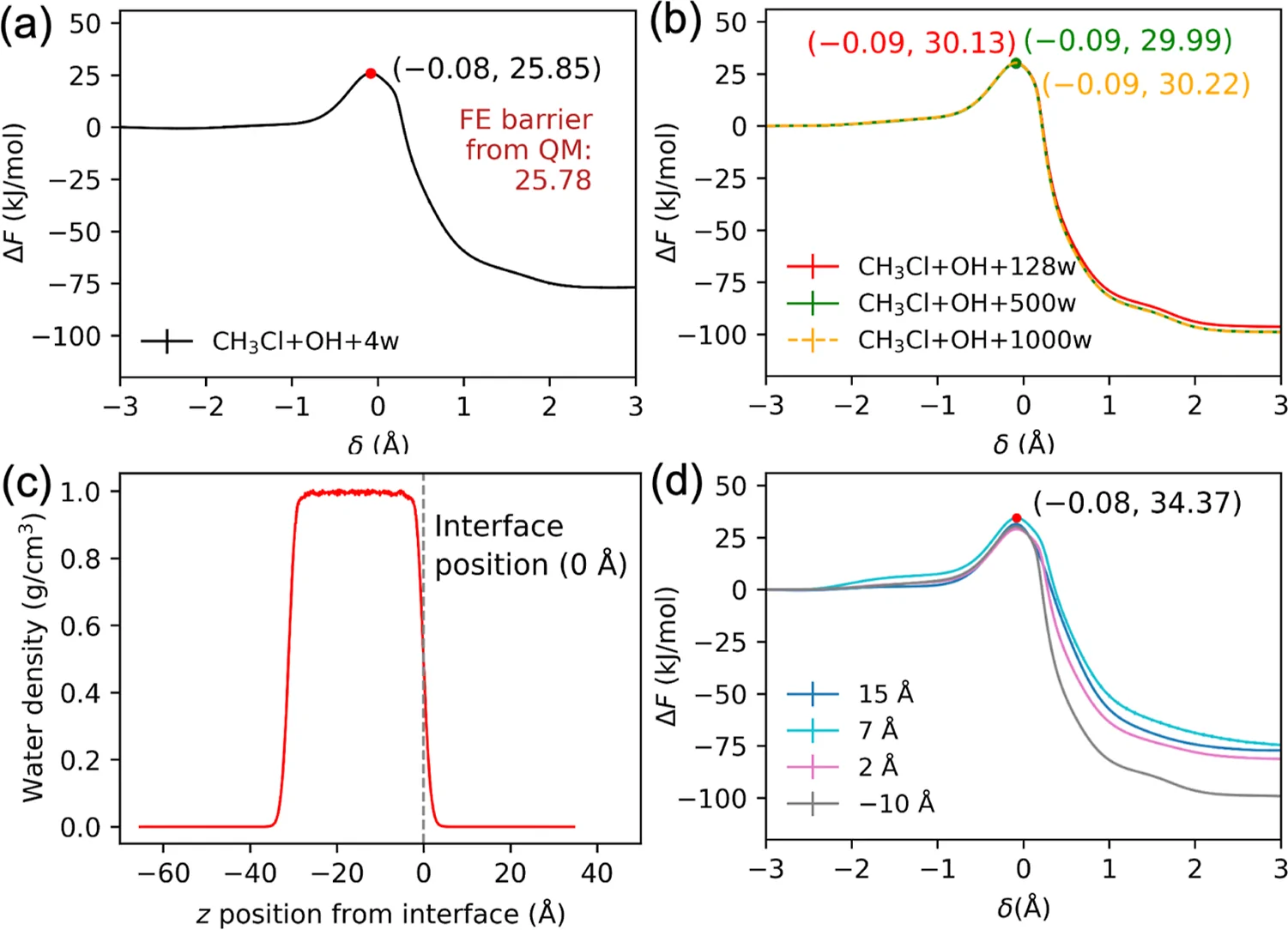
Reaction free energy profiles in the (a) gas phase, (b) bulk water, and (d) at selected *z* positions in a 1000-water slab system. Error bars (too small to be visible) were one standard deviation of the mean, estimated from 5 independent blocks of 30 pulls. Numbers in the parentheses are (δ, Δ*F*) at the barrier top (for *z* = 7 Å in (d)). (c) Water density profile and location of the Gibbs dividing surface (defined at 50% of the bulk water density) along the *z*-axis in a 1000-water slab system. We have summarized the TS positions, free energy barriers Δ*F* ^‡^, and errors in Table S8 in the Supporting Information.

For bulk water systems, the resulting reaction free energy profiles are shown in Figure 5b for bulk systems containing 128, 500, and 1000 water molecules. The barrier heights, obtained as the free energy difference between the TS and the state at δ = −3 Å, are all ∼30 kJ/mol and essentially independent of system size beyond 128 water molecules.

For the air–water interface systems, we first located the Gibbs dividing surface at 50% of the bulk water density, defining this as *z* = 0 (Figure 5c). To examine how the reaction barrier also depends on the distance from the interface, we restrained the transferred hydrogen H* at *z* = – 10, −6, −3, 0, 2, 4, 7, 10, and 15 Å. A primary motivation for these simulations is to investigate the intrinsic nature of the reaction across a fully hydrated environment, the interface, and gas-phase conditions. In particular, the results for large *z* values are relevant to the atmospheric CH_3_Cl + OH reaction in the gas phase when the air is dry, and there are no nearby aqueous aerosols.

The complete set of the resulting free energy profiles is shown in Figure S4 in the Supporting Information, and selected curves for *z* = −10, 2, 7, and 15 Å are presented in Figure 5d. In the following, we focus primarily on the region near and just above the interface, within a few Å of the Gibbs dividing surface, which is most relevant to interfacial reactivity.

For *z* < 0, the reaction site is fully immersed in the aqueous phase. The corresponding barriers are all close to 30 kJ/mol, in line with the results for bulk water systems in Figure 5b. The maximum barrier, ∼34.4 kJ/mol, occurs at *z* = 7 Å. As the reaction site moves from the bulk region toward the interface, the barrier decreases slightly. The lowest value, ∼29.0 kJ/mol, is observed at *z* = 2 Å, where the environment is interfacial but still strongly hydrated. In other words, there is a modest reduction in the reaction barrier at distances slightly above the interface, although there is a positive free energy cost to bring the reaction complex to such locations as we will show later.

For comparison, we also computed free energy profiles using quantum mechanics/molecular mechanics (QM/MM) simulations for a spherical droplet of 1000 water molecules. These QM/MM results are provided in the Supporting Information.

What is particularly interesting about these results is the systematic difference (about 3 kJ/mol) between the barriers at *z* = 15 Å and *z* = 7 Å, which reflects the distinct degrees of coupling to the liquid phase at these distances. Another contributing factor is the formation of a “water finger” (or water wire) connecting the reactants to the interface, especially at smaller *z*. An example of such a structure is shown in Figure 6. The discussion of water fingers is most often associated with liquid–liquid interfaces and much less so for the air–water interface.^64–68^

12 $$ECN=\sum_{i}^{N_{O}}s\left(r_{i}\right)$$

where *N*_O_ is the number of water oxygen atoms, *r*_*i*_ is the distance between oxygen *i* and H*, and *s*(*r*_*i*_) is a switching function defined by

13 $$s\left(r_{i}\right)=\{\begin{array}{ll} \frac{1-{\left(r_{i}/r_{0}\right)}^{n}}{1-{\left(r_{i}/r_{0}\right)}^{m}} & r_{i}\neq r_{0}\\ n/m & otherwise \end{array}$$

with *r*_0_ = 6 Å, *n* = 20, and *m* = 40. These parameters were chosen to differentiate between the coordinated and noncoordinated configurations. We note that the value of ECN is not the same as that of a typical integer coordination number when 0 < *s*(*r*_*i*_) < 1. We controlled the ECN by applying a harmonic potential with a force constant of 150 kJ/mol.

**Figure 6:**
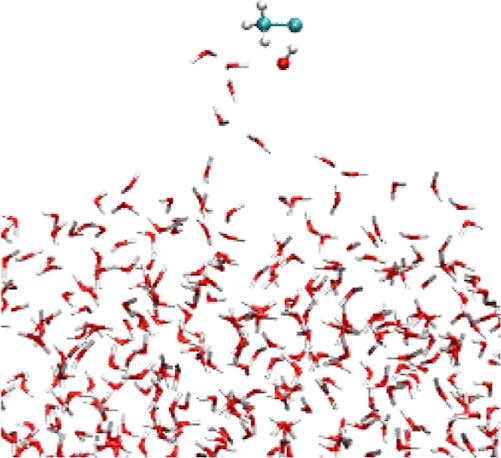
Representative snapshot showing a water finger connecting the reactant complex to the air–water interface. While the water fingers observed in this reaction are sometimes transient, their occurrence has prompted us to investigate the effect of hydration on the reaction barrier by explicitly controlling it. To control the degree of hydration, we applied enhanced sampling to control the effective coordination number (ECN) of the reactive hydrogen with respect to nearby water oxygen atoms.

By holding CH_3_Cl at *z* = 15, *z* = 10, *z* = 7, and *z* = 4 Å, we sampled different hydration states around the reactive hydrogen by controlling the ECN value between 0 and 4, roughly referring to 0 to 4 water neighbors, as shown in the snapshots in Figure 7. The resulting free energy profiles are shown in Figure 8. For ECN = 0, the free energy barriers at *z* = 15 and *z* = 10 Å are ∼32 kJ/mol, consistent with the results in Figures 5d and S4, in which the reactants were found to have no hydration. At *z* = 7 Å, the barrier was found to reduce from ∼38 kJ/mol at ECN = 0 to ∼35 kJ/mol at ECN = 2, in line with the barrier at the same *z* value without explicit ECN control (Figure 5d). This is a strong indication that water fingers, corresponding to nonzero ECN, are typically present at *z* = 7 Å even when ECN is not restrained, which is confirmed by trajectory inspection.

**Figure 7:**
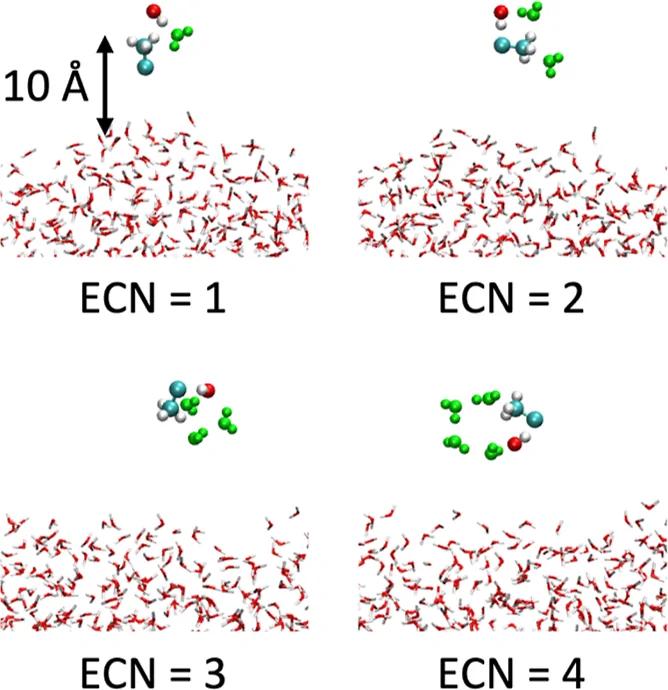
Snapshots of the reactants above the air–water interface in a slab system with effective coordination number (ECN) of water oxygens around the reactive H* restrained to 1, 2, 3, and 4, respectively, at *z* = 10 Å. Water molecules contributing to ECN are highlighted in green.

**Figure 8:**
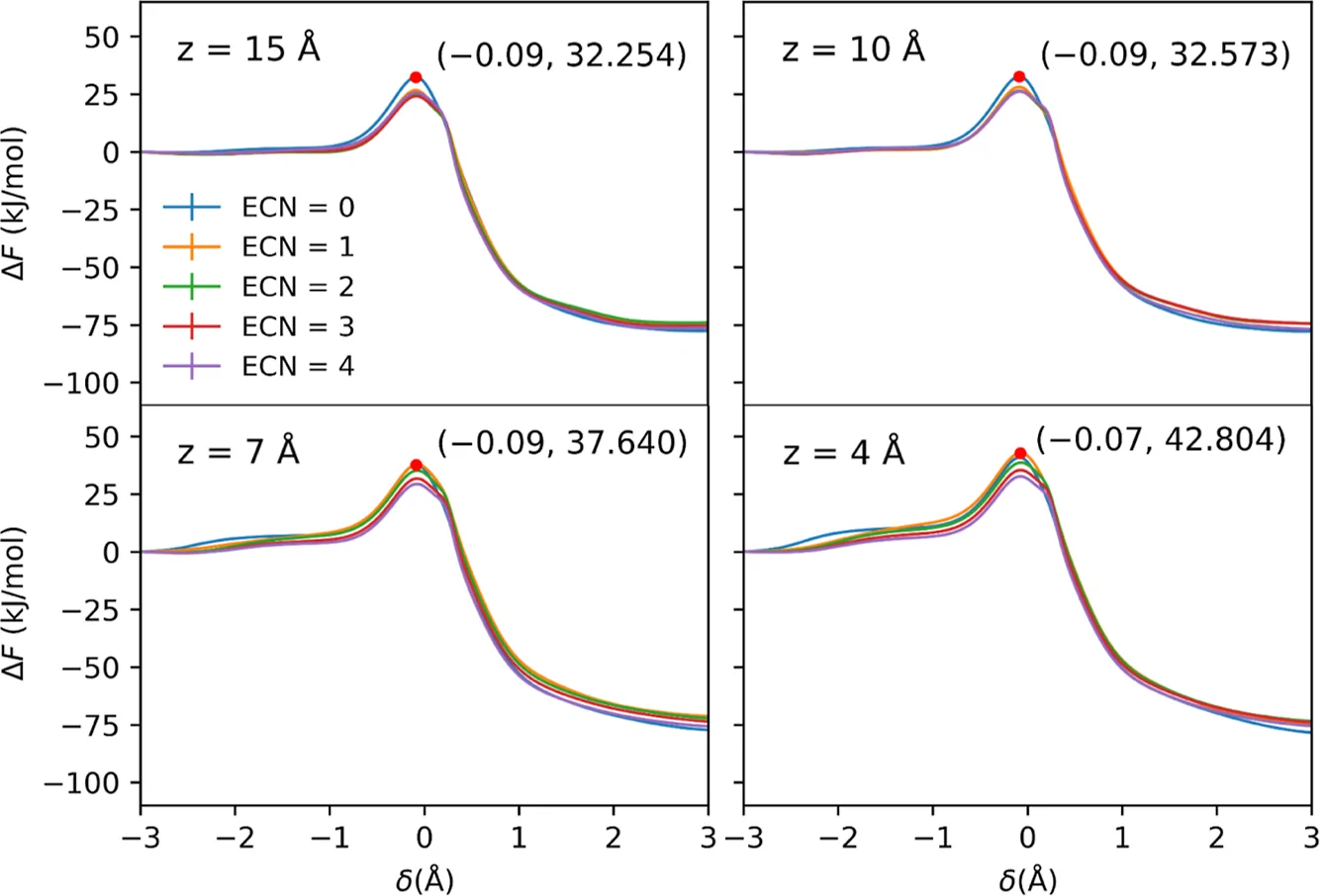
Reaction free energy profiles as a function of *z* and ECN. Refer to Figure 5 about the error estimates. Numbers in the parentheses are the (δ, Δ*F*) values for ECN = 0 (upper panels) and ECN = 1 (lower panels).

Overall, these results show that the reaction barrier decreases as the TS becomes more hydrated (increasing ECN), and such hydration is readily supplied near the interface, whereas it is much less accessible far out in the gas phase. The barrier reduction probably arises from stabilizing interactions between water molecules and the TS. This mechanism might share some details with oil–water interface systems, where dangling OH groups of water molecules can form hydrogen bonds that stabilize transition states.^69,70^ Also, a recent study by Xia et al.^71^ has proposed a related water-promoted mechanism at the air–water interface, where OH radicals preferentially attack the OH group of methanediols (HOCH_2_OH) instead of the exposed CH_2_ group, leading to faster oxidation because the OH group of the alcohol forms hydrogen bonds with the interfacial water molecules. Moreover, Mitroka et al.^72^ showed that the polarized TS of a similar hydrogen-abstraction reaction with OH radicals can be stabilized by hydrogen bonding with neighboring water molecules, whereas the reactivity decreases in dipolar aprotic solvents.

To make the dependence of the barrier on local hydration more transparent, we list the TS positions, Δ*F* ^‡^ values, and uncertainties as a function of both *z* and ECN in Table 1.

**Table 1:** Effect of the Effective Coordination Number (ECN) of Water Oxygens around the Reactive Hydrogen on the Interfacial Reaction Free Energy Barrier at Selected Distances *z* from the Air–Water Interface^a^

*z*/Å	ECN	TS position/Å	Δ*F* ^‡^/kJ mol^–1^	uncertainty/kJ mol^–1^
*z* = 15	0	–0.086	32.2541	±0.0264
	1	–0.089	26.6757	±0.0185
	2	–0.086	24.5140	±0.0247
	3	–0.083	24.0820	±0.0777
	4	–0.083	25.6586	±0.0531
*z* = 10	0	–0.085	32.5734	±0.0266
	1	–0.087	28.0701	±0.1003
	2	–0.085	26.4644	±0.0812
	3	–0.081	26.1619	±0.1044
	4	–0.081	26.3443	±0.0473
*z* = 7	0	–0.085	37.6397	±0.0611
	1	–0.071	37.8009	±0.0733
	2	–0.076	35.2181	±0.0794
	3	–0.076	31.7332	±0.0555
	4	–0.079	29.3880	±0.0559
*z* = 4	0	–0.084	40.8261	±0.0644
	1	–0.074	42.8041	±0.0737
	2	–0.069	38.6590	±0.0325
	3	–0.074	35.3856	±0.0617
	4	–0.075	32.6495	±0.0313

a One-standard-deviation uncertainties were obtained from block analysis of the ECN-restrained SMD trajectories.

### 3.4 Propensity of CH_3_Cl and OH Radical for the Air–Water Interface

Since the reaction rate depends on both the reaction free energy barrier and the local concentrations of the reactants, it is essential to compare them in the gas phase, at the air–water interface, and in the bulk water phase. We computed the solvation free energy profiles for CH_3_Cl and OH as functions of their distance from the interface using umbrella sampling (US) in both slab and droplet geometries. Here, we focus on the droplet results, and the slab data are provided in the Supporting Information. Overall, the two geometries yield similar profiles, with the slab showing slightly more negative solvation free energies.

Figure 9 shows the free energy profiles of solvating a single CH_3_Cl or OH radical into water droplets containing 500, 1000, or 2000 water molecules. In these simulations, a flat-bottom wall potential was applied at a radius of 60 Å from the droplet center (force constant 20 kJ/mol/Å^2^) to prevent the escape of the evaporated water molecules if any water oxygen is 60 Å farther away from the droplet center.

**Figure 9:**
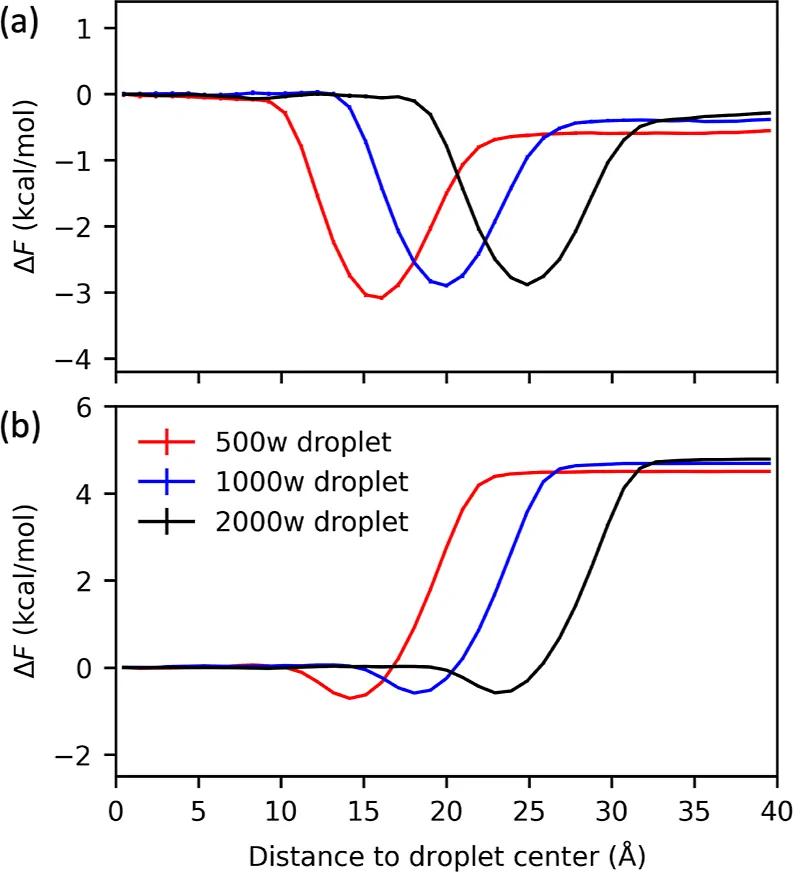
Free energy profiles for solvating (a) one CH_3_Cl or (b) one OH radical into droplets containing 500, 1000, or 2000 water molecules, as a function of the distance between the solute center of mass and the droplet center of mass. Error bars were obtained by bootstrapping.

As indicated by the wells in the free energy profiles near the droplet interface, CH_3_Cl has a clear preference for the air–water interface. This is consistent with the fact that organic species often accumulate and form coatings at aqueous interfaces, as reported by previous experimental and computational studies.^7,73–75^ In contrast, the OH radical exhibits a much weaker preference for the interface, as indicated by the shallower dips. As a side note, there have been different reported values of Δ*F*_solv_ for the OH radical. Hanson et al.^76^ derived a value of −3.9 kcal/mol from thermochemical cycles, while Henry’s law constants for OH span 25.3–9017.9 M/atm,^77^ implying Δ*F*_solv_ values between −1.9 and −5.4 kcal/mol. Therefore, our calculated values, between −4.8 and −4.5 kcal/mol, fall comfortably within this range.

We next examined how an interfacial CH_3_Cl coating affects the interfacial propensity of OH. In Figure 10, we compared two free energy profiles for one OH radical solvated into a 1000-water droplet in the presence or absence of 50 additional CH_3_Cl molecules forming an interfacial coating. The coating only slightly perturbs the profile, implying that the interfacial OH concentration is only modestly enhanced in the presence of a CH_3_Cl coating, with near monolayer coverage (see Supporting Information for a snapshot). A quantitative analysis of concentration and rate enhancement is presented in the next section.

**Figure 10:**
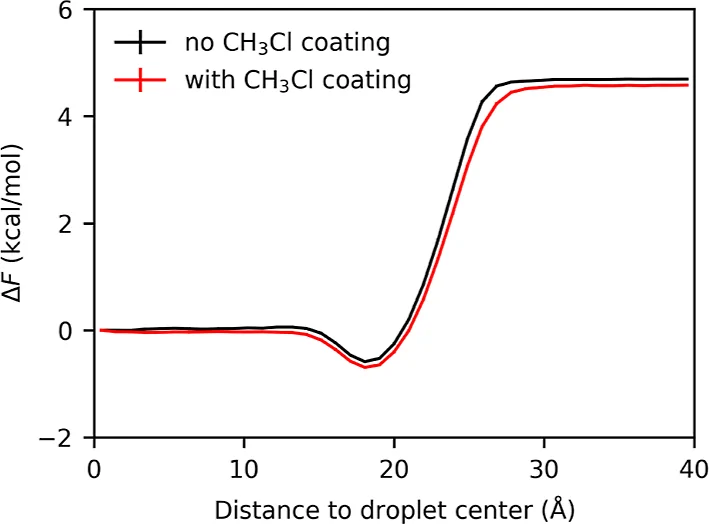
Free energy profiles of solvating one OH radical into a 1000-water droplet with and without 50 additional CH_3_Cl molecules forming a coating layer. Error bars were obtained by bootstrapping.

Although VSFG experiments by Harper et al.^78^ did not detect a clear CH_3_Cl signal at the air–water interface, their accompanying MD simulations showed that CH_3_Cl nevertheless adsorbs at the surface and exhibits enhanced interfacial number densities relative to both the gas phase and bulk water. They attributed the lack of VSFG intensity to a combination of low interfacial coverage, orientational disorder, and short residence times rather than to negligible adsorption. Our free–energy profiles, which predict an interfacial thermodynamic well for CH_3_Cl, are therefore consistent with the MD-inferred interfacial enrichment, while remaining compatible with the absence of a strong VSFG signature.

### 3.5 Concentration and Rate Enhancement at the Interface

With the obtained reaction free energy barriers Δ*F* ^‡^ and solvation free energies Δ*F*_solv_, we can estimate relative reactant concentrations and reaction rates in the gas phase, at the interface, and in bulk water. Within transition state theory, the rate constant is estimated from the Eyring equation^79,80^

14 $$k=\frac{\kappa k_{B}T}{h}\exp\left(-\Delta F^{‡}/RT\right)$$

where *k* is the rate constant, κ is the transmission coefficient, *k*_B_ is the Boltzmann constant, *R* is the ideal gas constant, and *h* is the Planck constant.

From the free energy profiles in Figure 5d, and assuming the same κ in all environments, the ratio of rate constants at 300 K is *k*_gas_: *k*_inter_: *k*_aq_ = 1:3.7:2.3. To quantify how the statistical uncertainty in Δ*F* ^‡^ affects the rate constants, we propagated the errors from the free energy calculations through eq 14. Detailed error propagation is provided in the Supporting Information. Representative barriers, associated uncertainties, and the corresponding relative rate expressions and error factors are summarized in Table 2. The small barrier uncertainties reported in Tables 1 and S8 translate into modest multiplicative uncertainties in the corresponding rate constants.

**Table 2:** Representative Reaction Free Energy Barriers, Relative Rate Expressions, and Propagated Rate Uncertainties at 300 K for the CH_3_Cl + OH Reaction in the Gas Phase, Bulk Water, and at the Air–Water Interface^a^

environment	Δ*F* ^‡^/kJ mol^–1^	*k*/*k*_gas_ ^b^	*f*_*k*_ ^c^
gas (no water)	32.25 ± 0.03	1	1.01
bulk water	30.22 ± 0.06	≈2.3	1.02
interface	29.00 ± 0.02	≈3.7	1.01

a The gas-phase rate constant *k*_gas_ is used as the reference. Δ*F*_gas_^‡^ denotes the gas-phase barrier.

b Relative rates obtained from Eq 14 by referencing to the gas-phase barrier.

c Multiplicative rate uncertainty factor defined in the Supporting Information; for the small $\sigma_{\Delta F^{‡}}$ values reported in Tables 1 and S8, *f*_*k*_ is close to unity.

Relative equilibrium concentrations along the solvation coordinate are obtained from

15 $$\frac{c_{1}}{c_{2}}=\exp\left(-\Delta F_{solv}/RT\right)$$

where *c*_1_ and *c*_2_ are the local concentrations of a given species at two positions (1 and 2) along the coordinate, and Δ*F*_solv_ is the corresponding free energy difference. Figure 11 summarizes the analysis based on the 1000-water droplet data. Interfacial concentrations were estimated from the depths of the wells in Figure 9. According to our results, regardless of whether a CH_3_Cl coating is present, the overall reaction rate at the interface is predicted to be more than 2 orders of magnitude higher than in bulk water and more than 6 orders of magnitude higher than in the gas phase. This substantial rate enhancement arises predominantly from the strong accumulation of CH_3_Cl (and to a lesser extent OH) at the air–water interface, rather than from a large reduction of the reaction barrier itself. This picture is consistent with the broader mechanistic understanding of aqueous interfacial reactivity, which emphasizes the importance of interfacial enrichment and modified local environments in driving rate enhancements.^6–8^

**Figure 11:**
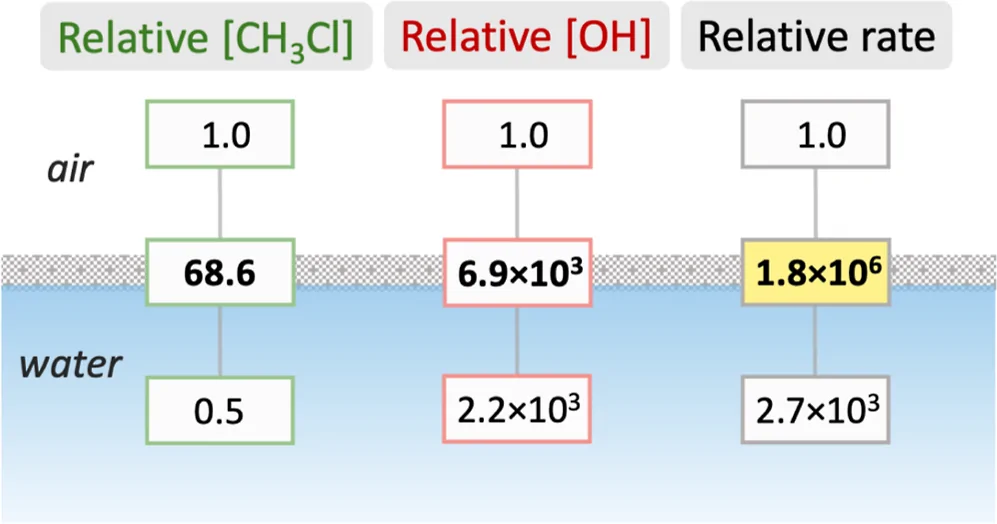
Relative concentrations of the reactants in bulk water, at the interface, and in the gas phase, and corresponding relative reaction rates. These concentration estimates were obtained from the solvation free energy profiles in Figure 9. The gas phase concentration and rate constant *k*_gas_ are normalized to 1.

What our work adds is a quantitatively calibrated, reaction-specific decomposition of these contributions for an atmospherically relevant radical reaction. By combining an EVB model parametrized against high-level ab initio data with converged free energy profiles in bulk and interfacial environments, we explicitly separate the effects of barrier modulation and interfacial partitioning on the rate of the reaction between CH_3_Cl and OH. Within this unified framework, we show that even a typically modest interfacial free energy well can generate more than 2 orders of magnitude enhancement in the effective interfacial rate relative to the aqueous bulk and over 6 orders of magnitude relative to the gas phase.

## 4 Conclusions

In this work, we investigated the reaction between methyl chloride (CH_3_Cl) and hydroxyl radicals (OH) near the air–water interface, a critical process in atmospheric chemistry. We developed a two-state reactive EVB model for this reaction, parametrized against high-level QM reference data, and combined it with a flexible polarizable water force field. This approach enabled us to obtain converged free energy profiles for the CH_3_Cl + OH hydrogen-abstraction reaction in bulk water and near the air–water interface.

In line with previous work on microdroplets and atmospheric aqueous interfaces, which has identified the enhanced interfacial concentrations as a key driver of accelerated reactions,^6–8^ we find that the reaction between CH_3_Cl and OH experiences only a modest reduction in the reaction free energy barrier at the interface. The dominant contribution to the interfacial rate acceleration instead arises from the thermodynamic accumulation of the surface-active reactants, which substantially increases their local concentrations.

Within the present EVB framework, we can quantify these two effects on equal footing, providing a concrete, quantitatively validated example of how barrier modulation and partitioning jointly control on-water reactivity for an atmospherically relevant radical reaction. By combining well-converged solvation free energies with interfacial and bulk free energy barriers, we demonstrate that the effective reaction rate at the interface is more than 2 orders of magnitude higher than that in the aqueous bulk and over 6 orders of magnitude higher than that in the gas phase.

The present EVB model is reaction-specific and was parametrized for the CH_3_Cl + OH hydrogen-abstraction reaction. We do not claim that the same parameters are directly transferable to other reaction families. Rather, our main methodological contribution is to provide a computationally tractable framework for building reaction-specific EVB models at aqueous interfaces, which can be extended to other environmental reactions by repeating the same parametrization and validation protocol against high-level reference data. Looking ahead, an important extension of this work will be to analyze the explicit local electric fields experienced along the reaction coordinate, for example, by projecting microscopic forces or fields onto the CV δ, in order to relate interfacial field fluctuations more directly to barrier modulation; such an analysis is beyond the scope of the present study.

In addition to EVB, machine-learned interatomic potentials (MLIPs) can be used as an alternative to model the same reaction and related interfacial processes.^81,82^ MLIPs can, in principle, reproduce complex reactive potential energy surfaces with high fidelity and remove the need for predefined empirical functional forms, such as the EVB coupling terms. This flexibility, however, typically comes with a much larger number of adjustable parameters and a possibly greater demand for more reference data. In this work, we deliberately adopted a relatively minimal EVB representation that captures the key reaction chemistry with a small number of empirical parameters. Because of its simple functional form, an EVB model is more computationally efficient and may also be more robust when the sampled configurations deviate substantially from the training set, whereas MLIPs can be less stable during extrapolation. Therefore, the choice between an EVB description and an MLIP may be viewed as a trade-off among simplicity, model flexibility, and robustness.

Finally, from an atmospheric perspective, these results highlight that the air–water interface can substantially accelerate the chemical transformation of surface-active species such as CH_3_Cl, beyond what bulk-phase kinetics alone would suggest.

## Supplementary Material





## Data Availability

Input files containing the force field/EVB parameters, analysis scripts, numerical data for all figures, and selected MD trajectories are available on GitHub (https://github.com/tsegroupcuhk/ch3cl-oh-evb).

## Notes

Cartesian coordinates of important stationary points in the gas phase, benchmark results for different quantum chemical methods, IRI analysis along IRC (including a video), force field parameters for the water, reactant and product models, off–term parameters of the EVB model, results for matching binding energy, Gibbs free energy estimation from potential energy, comparison of the solvation structure of OH radical in bulk water and at the interface, free energy profiles of the reaction fixed at different *z* locations of the air–water interface, QM/MM simulations, near monolayer coverage of CH_3_Cl on water droplet, thermodynamic limit of solvation free energies, free energy study of CH_3_Cl and OH sorption by water slabs, error propagation from free energy to rate constant (PDF)
A snapshot of IRI analysis on the TS is shown in Figure S1. The whole animation is provided in a Supplementary Video S1 (MP4)
