# Supplementary material for: Accelerated OH Oxidation of Methyl Chloride via Surface Accumulation at the Air–Water Interface
Source: ACS Phys Chem Au. 2026 Aug 19;6(5):1252–63. doi: 10.1021/acsphyschemau.6c00076 (PMC13614031; doi:10.1021/acsphyschemau.6c00076)
Supplement: Supplementary file 1 [file pg6c00076_si_001.pdf]

Supporting Information

Accelerated OH Oxidation of Methyl Chloride via Surface Accumulation at the Air-Water Interface

Yutong Yang,[1] Chung Chi Chio,[1] and Ying-Lung Steve Tse[1]*

[1]Department of Chemistry, The Chinese University of Hong Kong, Sha Tin, New Territories, Hong Kong, China

*Corresponding author. E-mail: stevetse@cuhk.edu.hk

Table of Contents

Cartesian coordinates of important stationary points in the gas phase 2

Benchmark results for quantum chemical methods 5

IRI analysis along IRC 6

Force field parameters for water, reactant, and product models (Tables S2-S5)....7

EVB off-term parameters 10

Results for matching binding energy 11

Estimate Gibbs free energy from potential energy....11

Bulk vs interfacial O–O* radial distribution functions....12

Free energy profiles of the reaction fixed at different *z* locations of the air-water interface13

Reaction free energy barriers and uncertainties in gas and condensed phases....14

QM/MM simulations 14

Near monolayer coverage of 50 CH_3Cl molecules on a water droplet....17

Thermodynamic limit of solvation free energies....17

Free energy study of CH_3Cl and OH sorption by water slabs....19

Error propagation from free energy to rate constant 21

References....22

Cartesian coordinates of important stationary points in the gas phase

This section presents the Cartesian coordinates and electronic energies of the key stationary points on the gas-phase potential energy surface for the reaction between CH_3Cl and OH in the presence of four water molecules. These data allow full reproducibility of the benchmark potential energy surface used to parameterize the EVB model.

All geometries were optimized at the M06-2X-D3[1,2]/ma-def2-TZVP[3,4] level of theory and were reported in Å. For each point along the IRC, including the transition state (TS) structure, single-point energies were computed at the TightPNO DLPNO-CCSD(T)[5–9]/aug-cc-pVQZ[10] level. Frequency calculations at the M06-2X-D3/ma-def2-TZVP level were used to confirm the nature of each stationary point. Energies are reported in Hartree.

Reactant complex

Number of imaginary frequencies (M06-2X-D3/ma-def2-TZVP): 0 (minimum)

```
E(M06-2X-D3/ma-def2-TZVP) = -881.6122037
E(DLPNO-CCSD(T)/aug-cc-pVQZ) = -880.7845179
C        2.74405600      0.61884000     -0.00759300
H        1.94745500      0.55239500     -0.74348300
H        3.61614000      1.11041300     -0.42559000
H        2.40834300      1.12818200      0.89037400
Cl       3.22757400     -1.04364500      0.44340400
O       -0.09358500     -0.12466700     -1.86625900
H       -0.09217500     -0.71123300     -1.08949300
H       -0.24212700     -0.67704000     -2.63770100
O       -2.76444200     -1.10133400      0.35132700
H       -3.41752800     -1.50585100      0.92653200
H       -1.89315200     -1.42819000      0.62698300
O       -1.85875700      1.46729500     -0.35684600
H       -1.37089800      1.14861500     -1.13392200
H       -2.42509000      0.71489500     -0.11306300
O        0.04028200      1.46255100      1.49505200
H       -0.70622200      1.61257900      0.83355200
H        0.08310000     -0.33752900      1.17703100
O       -0.01538700     -1.20757000      0.73792900
H        0.81241100     -1.67870500      0.89176900
```

Transition state (TS)

Number of imaginary frequencies (M06-2X-D3/ma-def2-TZVP): 1 (The corresponding normal mode describes H-transfer between carbon and oxygen.)

```
E(M06-2X-D3/ma-def2-TZVP) = -881.6013379
E(DLPNO-CCSD(T)/aug-cc-pVQZ) = -880.7729732
C       1.89681800    1.01755800   -0.17470200
H       1.38385800    1.02391100   -1.13056100
H       2.61947100    1.81727100   -0.04842800
H       1.09973700    1.14105400    0.67609200
Cl      2.66667600   -0.54021100    0.07803100
O      -0.39425600   -0.33947500   -1.92251900
H      -0.43498100   -0.90664600   -1.13202500
H      -0.65779100   -0.88098800   -2.67051800
O      -3.29098300   -0.70824200    0.30396000
H      -3.96683400   -0.92472800    0.95010700
H      -2.48623500   -1.18687700    0.56105300
O      -1.84204500    1.59373100   -0.44831200
H      -1.43199500    1.13661000   -1.20021200
H      -2.56825200    1.00194600   -0.18867700
O      -0.02197900    1.12340500    1.49769700
H      -0.68569100    1.46728800    0.83506600
H      -0.32495500   -0.57394500    1.12370400
O      -0.60876200   -1.41222600    0.68985600
H       0.00346400   -2.09421200    0.98062800
```

Product complex

Number of imaginary frequencies (M06-2X-D3/ma-def2-TZVP): 0 (minimum)

```
E(M06-2X-D3/ma-def2-TZVP) = -881.6406033
E(DLPNO-CCSD(T)/aug-cc-pVQZ) = -880.8134256
C       2.21957800     -1.07245600      0.08856200
H       1.43323900     -1.27552600      0.79845200
H       2.86310300     -1.82588100     -0.33429500
H       0.64097400     -0.57439800     -1.98087700
Cl      2.78716300      0.52968200      0.01300300
O      -0.47137800     -0.67815200      1.84890000
H      -0.50735800      0.19900900      1.42375200
H      -0.85250900     -0.57997500      2.72501000
O      -3.31424100      0.80072300      0.14898700
H      -3.95718600      1.30590900     -0.35381600
H      -2.48289000      1.30375800      0.13056900
O      -1.94362200     -1.61200800     -0.41635300
H      -1.48922600     -1.60319900      0.44092600
H      -2.64947800     -0.95268700     -0.30733300
O      -0.21714200     -0.14810500     -1.89455100
H      -0.82513500     -0.81807100     -1.50268600
H      -0.34638400      1.00194700     -0.67759600
O      -0.61077900      1.51804700      0.12864300
H      -0.01984900      2.27301100      0.18994700
```

Benchmark results for quantum chemical methods

The reference forward reaction barrier in potential energy was calculated with TightPNO DLPNO-CCSD(T)[5–9]/aug-cc-pVQZ[10] as mentioned in the main text. We also conducted benchmark studies comparing some DFT functionals, such as BLYP,[11–13] B3LYP,[11,12,14,15], and ωB97M-V.[16] Both BLYP and B3LYP cannot capture the forward reaction barrier effectively. Please refer to Table S1 for a detailed comparison. The coordinates obtained from IRC were calculated at M06-2X-D3/ma-def2-TZVP level.

Table S1. Benchmark study on the reaction between CH_3Cl and OH radical with four water molecules nearby. The forward reaction barrier is evaluated along the IRC.

Single point calculation level	Potential energy surface along IRC	Forward reaction barrier (kJ/mol)
BLYP-D3/TZVP-GTH	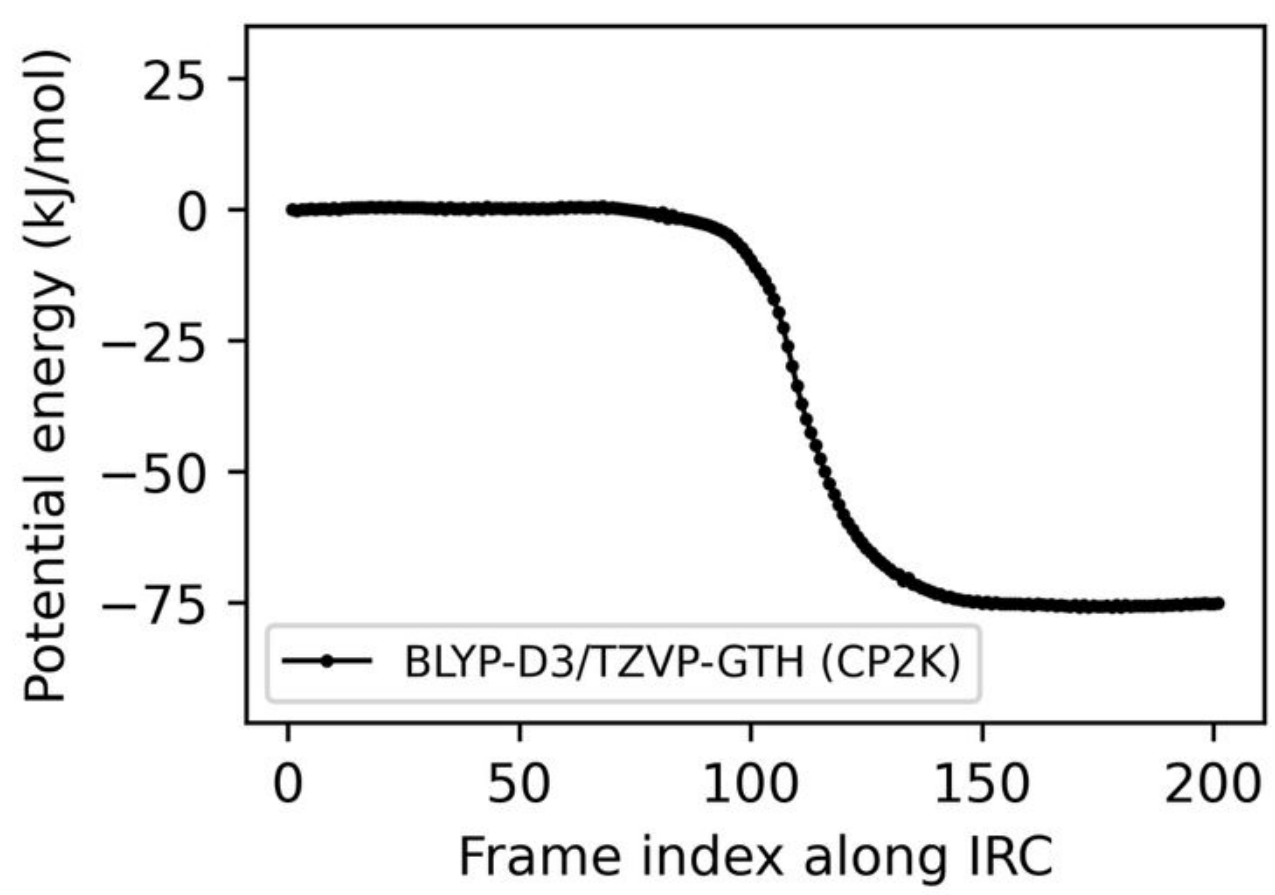	/
B3LYP-D3(BJ)/DZVP-MOLOPT-SR-GTH	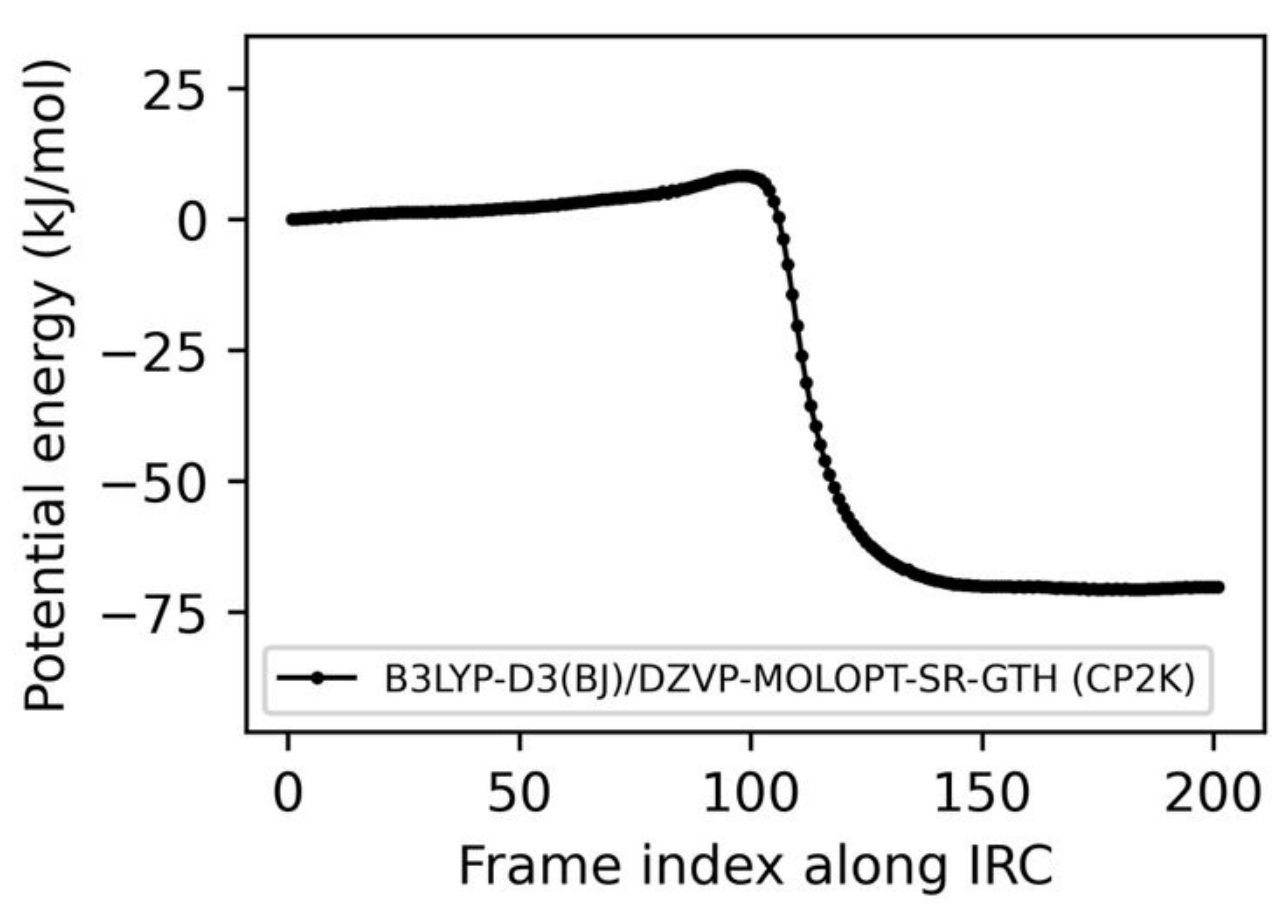	8.237

ωB97M-V/ma-def2-TZVP	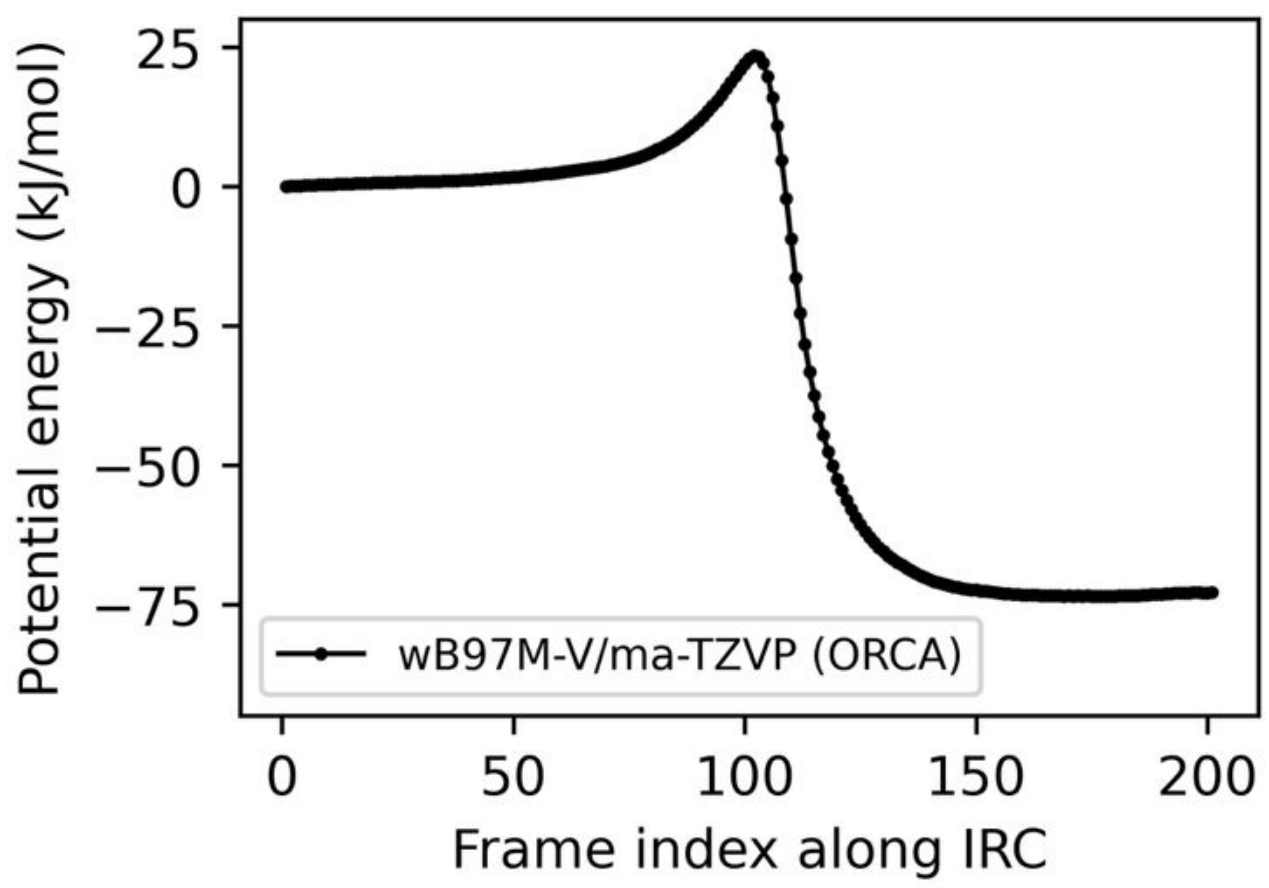	23.423
TightPNO DLPNO-CCSD(T) /aug-cc-pVQZ	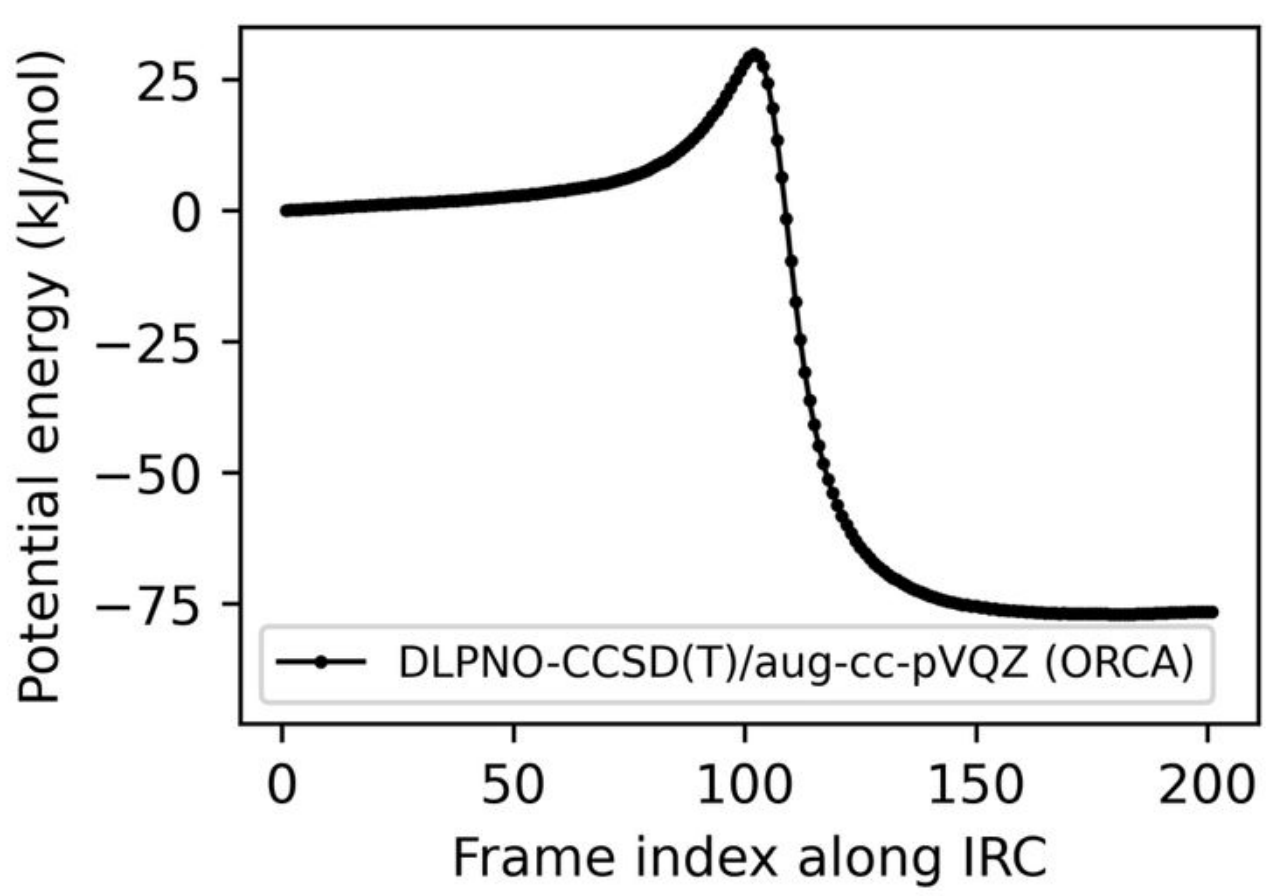	**29.852**

IRI analysis along IRC

We also analyze the electron structure characteristics along the IRC to obtain more valuable information on the chemical reaction. To show the changes of both bond interaction and weak interactions along the reaction course, the interaction region indicator (IRI) analysis,[17] which was proposed recently, was implemented using Multiwfn.[18,19] In the IRI representation, all types of interactions, including chemical bonds and weak interaction regions, can be displayed intuitively and clearly in the same plot.

A snapshot of IRI analysis on the TS is shown in Figure S1. The whole animation is provided in a Supplementary Video S1. From this figure, we can see that at the TS, a chemical bond is ready to form between the reactive hydrogen and the oxygen of the OH radical.

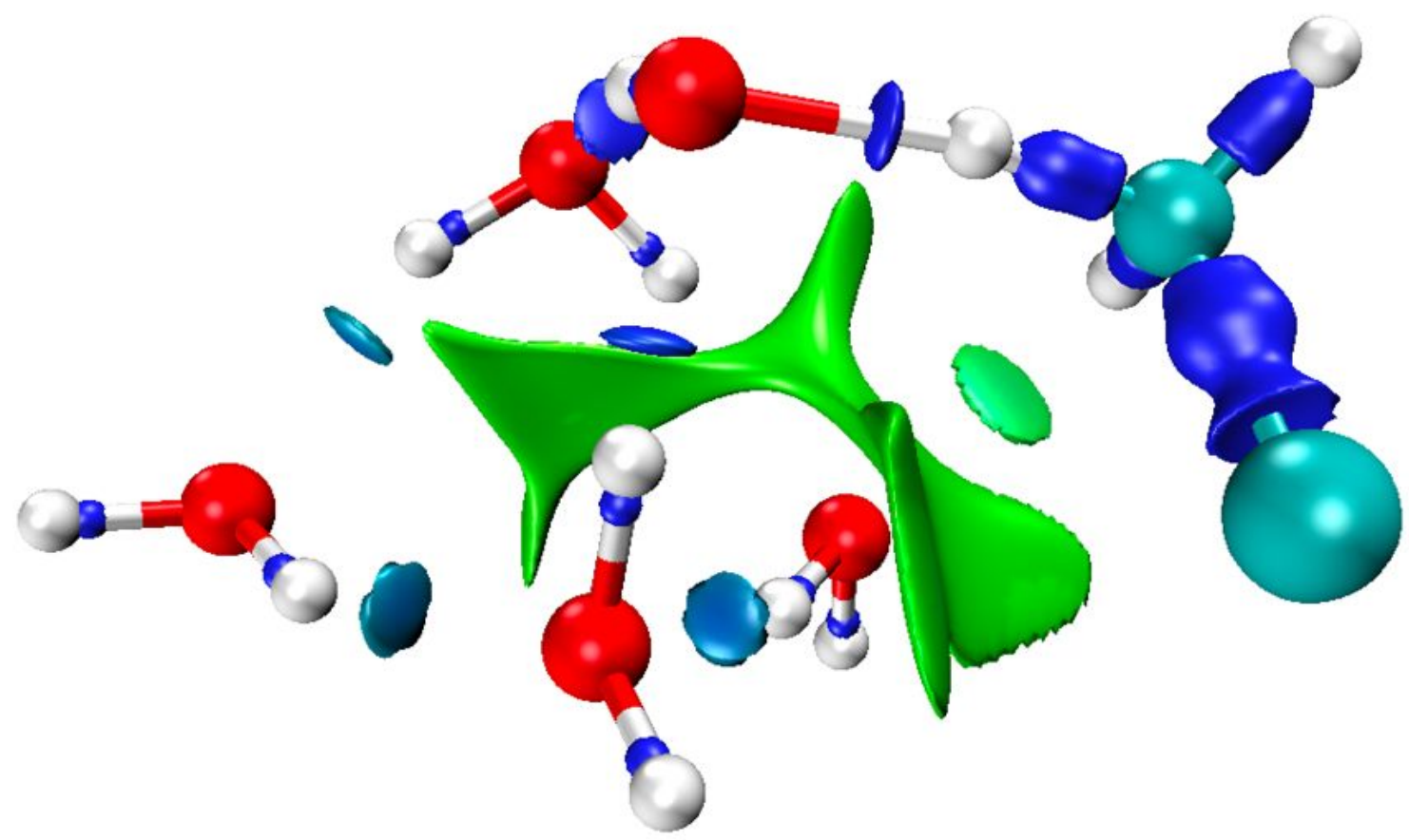

Figure S1. IRI snapshot of the transition state of the reaction with four additional water molecules.

Force field parameters for water, reactant, and product models (Tables S2-S5)

All the parameters were fitted to reproduce the potential energy surface along the IRC of the reference reaction, the binding energies of each substrate with one to four water molecules, and the first peak height of the O–O* RDF. In Tables S2-S5, WAT is for water, OHR is OH radical, MEC is CH_3Cl, and MER is CH_2Cl radical.

Table S2. Atomic masses *M*, partial charges *Q*, polarizability volumes α', and scaling factors THOLE[20] of different atom types of the optimized EVB model. Polarization of all atoms (except for hydrogen and virtual atoms) is modeled with Drude oscillators.[21–24] The polarizability α can be calculated by $\alpha = 4\pi\varepsilon_0\alpha'$, and the Drude charges q_D are always negative.

	Atom type	*M*	*Q*	α' / Å^3	THOLE
**WAT**	ODW	15.9994	0.00000	0.87641	1.3
	HDW	1.0080	0.53242	/	/
	LPDW	0.0000	−1.06484	/	/
**OHR**	OOHR	15.9994	−0.41554	1.03756	1.3
	HOHR	1.0080	0.41554	/	/

	LPOH	0.0000	0.00000	/	/
**MEC**	C3MC	12.0100	−0.32300	1.59686	1.280
	H1MC	1.0080	0.19877	/	/
	CLMC	35.4500	−0.27331	1.93495	1.280
**MER**	C3MR	12.0100	−0.35745	1.60850	1.280
	H1MR	1.0080	0.26131	/	/
	CLMR	35.4500	−0.16517	1.90466	1.280
**All Drude oscillators**	DRUD	0.4000	$\alpha = q_D^2/k_D$	/	/

Table S3. Bond parameters of the optimized EVB model. k_b is the harmonic bond force constant, r_0 the equilibrium bond length. "X"" denotes a generic atom in bonds involving Drude oscillators.

	**Atom type 1**	**Atom type 2**	k_b **/ kcal·mol^{-1}·Å^{-2}**	r_0**/ Å**
**WAT**	ODW	HDW	Refer to SWM4/Fw[25]	0.945700
**OHR**	OOHR	HOHR	547.062	1.022378
**MEC**	C3MC	H1MC	302.308	1.089777
	C3MC	CLMC	310.702	1.761591
**MER**	C3MR	H1MR	247.521	1.074258
	C3MR	CLMR	209.087	1.671253
**All Drude oscillators**	X	DRUD	k_D = 500.0	0.000000

Table S4. Angle parameters of the optimized EVB model. k_a is the harmonic angle force constant, θ_0 the equilibrium angle.

	Atom type 1	Atom type 2	Atom type 3	k_a / kcal·mol^{-1}·rad^{-2}	θ_0/ deg
**WAT**	HDW	ODW	HDW	Refer to SWM4/Fw[25]	108.500
**MEC**	H1MC	C3MC	H1MC	55.9342	111.449
	H1MC	C3MC	CLMC	40.7907	111.252
**MER**	H1MR	C3MR	H1MR	31.2589	125.810
	H1MR	C3MR	CLMR	32.8700	116.012

Table S5. Lennard-Jones parameters of the optimized EVB model. ε is the well depth and R_{min} $=2^{1/6}\sigma$ the distance at the potential minimum. “X” is a wild card.

	Atom type 1	Atom type 2	ε/ kcal·mol^{-1}	$R_{min}=2^{1/6}\sigma$/Å
**WAT-WAT**	ODW	ODW	0.198476	3.586232
	ODW	HDW	0.000000	1.793116
	HDW	HDW	0.000000	0.000000
**WAT-OHR**	ODW	OOHR	0.179846	3.509825
	ODW	HOHR	0.015418	2.740121
	HDW	OOHR	0.100350	2.110210
	HDW	HOHR	0.077192	2.091058
**WAT-MEC**	ODW	C3MC	0.081147	3.789933
	ODW	H1MC	0.003755	1.881383
	ODW	CLMC	0.000323	3.207556
	HDW	C3MC	0.001128	2.746497
	HDW	H1MC	0.007352	1.703672
	HDW	CLMC	0.076591	3.154352
**WAT-MER**	ODW	C3MR	0.274509	3.604560

	ODW	H1MR	0.047402	2.487663
	ODW	CLMR	0.284055	3.667240
	HDW	C3MR	0.035214	2.323001
	HDW	H1MR	0.000128	2.416658
	HDW	CLMR	0.063687	3.394034
**OHR-MEC**	OOHR	C3MC	0.441016	3.424730
	OOHR	H1MC	0.000043	2.199109
	OOHR	CLMC	0.286452	3.657409
	HOHR	C3MC	0.042525	2.013923
	HOHR	H1MC	0.009156	1.764497
	HOHR	CLMC	0.082345	3.394034
**All interactions with Drude and water/OHR virtual site**	X	DRUD	0.000000	0.000000
	X	LPDW	0.000000	0.000000
	X	LPOH	0.000000	0.000000

EVB off-term parameters

Table S6. The parameters in the off-diagonal coupling term in the EVB Hamiltonian and the energy difference V between the two EVB states. The functional form follows the expression described in the main text.

D **(kJ/mol)**	***A*** **(Å^{-2})**	***d_0*** **(nm)**	***a*** **(Å^{-2})**	***b*** **(Å^{-1})**	***c***	***V*** **(kJ/mol)**
119.851809	242.222362	0.010177	4.879339	−5.678966	1.461855	−64.741769

Results for matching binding energy

Table S7. Binding energies (BE) for solutes complexed with *n* water molecules. Reference values are from DLPNO-CCSD(T)/aug-cc-pVQZ with counterpoise correction for the basis set superposition error (BSSE). [26,27]

System	Reference BE (kJ/mol)	Predicted BE (kJ/mol)	BE difference (kJ/mol)	Relative difference
**CH_3Cl-1w**	−14.71760921	−13.63408238	−1.08352683	7.3621%
**CH_3Cl-2w**	−44.66211777	−42.32539554	−2.33672223	5.2320%
**CH_3Cl-3w**	−57.19929877	−53.49217757	−3.70712120	6.4811%
**CH_3Cl-4w**	−108.06221600	−102.43014188	−5.63207412	5.2119%
**OH-1w**	−22.90770037	−21.01470056	−1.89299981	8.2636%
**OH-2w**	−60.21384838	−50.05995135	−10.15389703	16.8631%
**OH-3w**	−105.52094510	−91.69922800	−13.82171710	13.0986%
**OH-4w**	−128.22301190	−105.87776310	−22.34524880	17.4269%
**CH_2Cl-1w**	−11.37731886	−11.51571588	0.13839702	1.2164%
**CH_2Cl-2w**	−40.05239817	−41.08399790	1.03159973	2.5756%
**CH_2Cl-3w**	−76.07808983	−75.59481917	−0.48327066	0.6352%
**CH_2Cl-4w**	−122.33404220	−123.01667017	0.68262797	0.5580%

Estimate Gibbs free energy from potential energy

For the gas-phase reaction with four explicit water molecules, Gibbs free energies along the intrinsic reaction coordinate (IRC) were estimated using the Shermo code[28] by combining the DLPNO reference electronic energies with thermal corrections derived from the M06-2X-D3 frequency calculations. The resulting free energy profile is plotted as the orange points in Figure S2, while the corresponding QM potential energy surface is shown as black points. Applying the thermal correction to the free energy reduces the forward barrier slightly to 26 kJ/mol, compared with the potential energy barrier of 30 kJ/mol. This barrier height agrees with the EVB result reported in the main text.

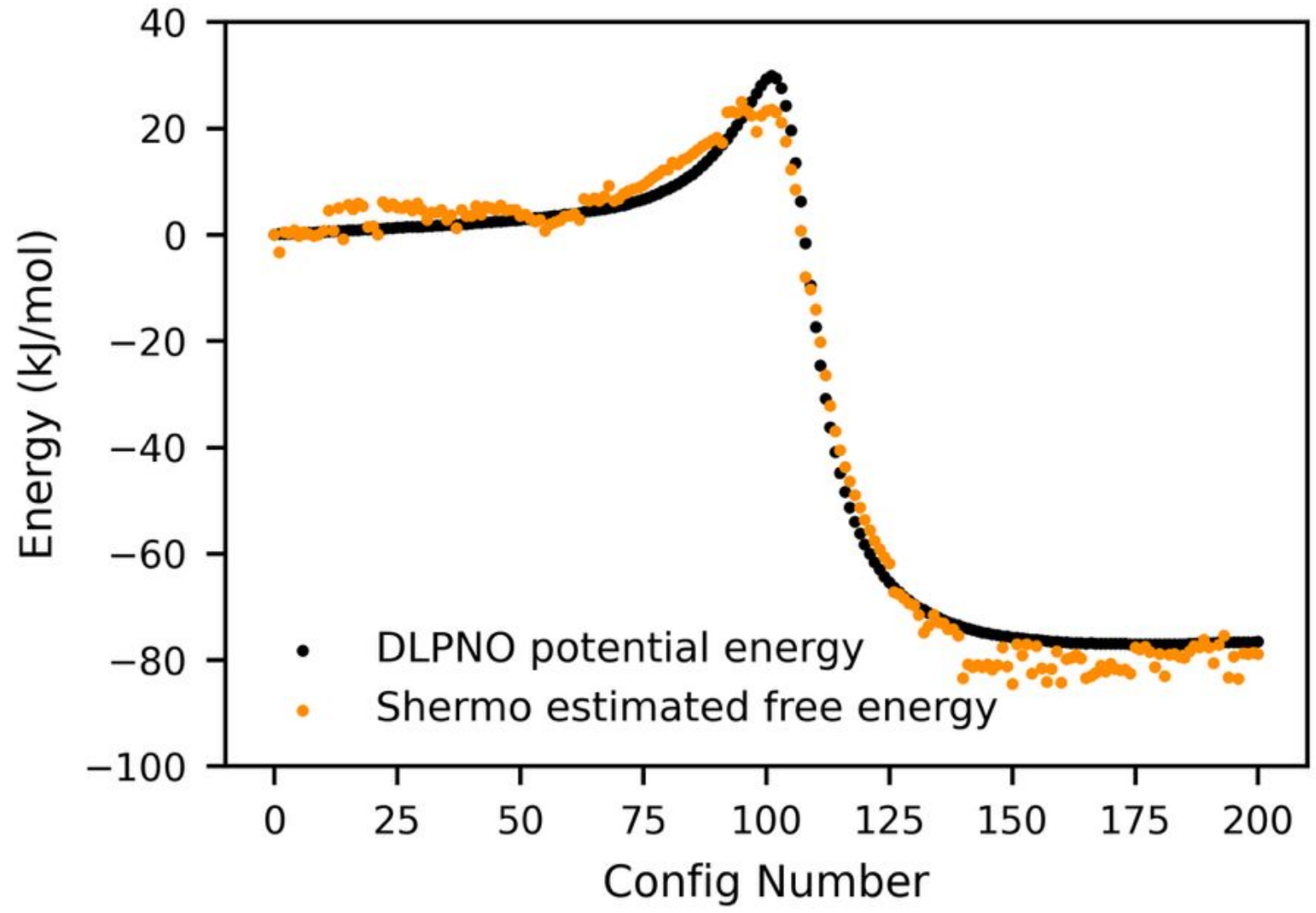

Figure S2. Gibbs free energies (orange points) estimated from the reference DLPNO potential energies (black points) for 201 configurations along the IRC. The system comprises the reactant molecules plus four explicit water molecules.

Bulk vs interfacial O–O* radial distribution functions

Figure S3 compares the O–O* (water oxygen – hydroxyl oxygen) radial distribution function $g_{\mathrm{O-O^*}}(r)$ in bulk liquid water with an analogous curve computed for an air–water slab, both using the same EVB Hamiltonian. The position of the first peak is very similar in the two cases, but the interfacial peak is lower, and the corresponding coordination number is reduced because part of the hydroxyl oxygen coordination shell extends into the vapor phase. Due to the anisotropy of the interface and the use of the bulk oxygen density for normalization, the interfacial RDF does not approach 1 at large r; instead, its tail remains below unity, reflecting the lower average local water density around OH near the interface.

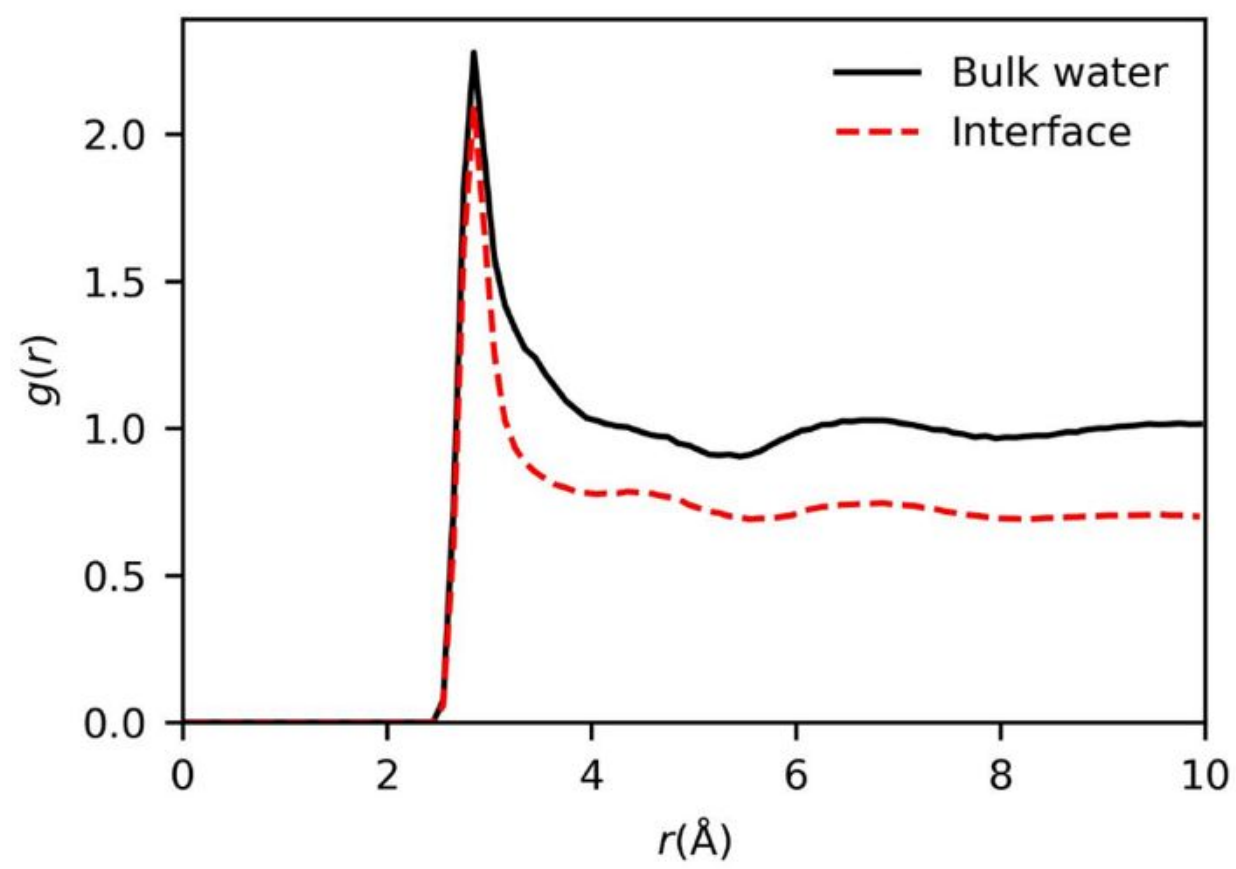

Figure S3. Comparison of O–O* radial distribution functions in bulk liquid water and at the air–water interface, computed with the same EVB Hamiltonian. The bulk curve is the same as in Figure 4(a) in the main text, while the interfacial curve is obtained from a 1000-water slab system.

Free energy profiles of the reaction fixed at different *z* locations of the air-water interface

To understand the impact of the reaction site's location relative to the interface, we sampled the reaction at several different *z*-distances from the interface. We choose ten distances, which are z = –10, –7, –4, –2, 0, 2, 4, 7, 10, 15 Å. The reaction occurs in the aqueous phase when z is negative and takes place in the air when z is positive. For each z value, we did 150 SMD simulations to estimate the free energy profile, and the results are shown in Figure S4.

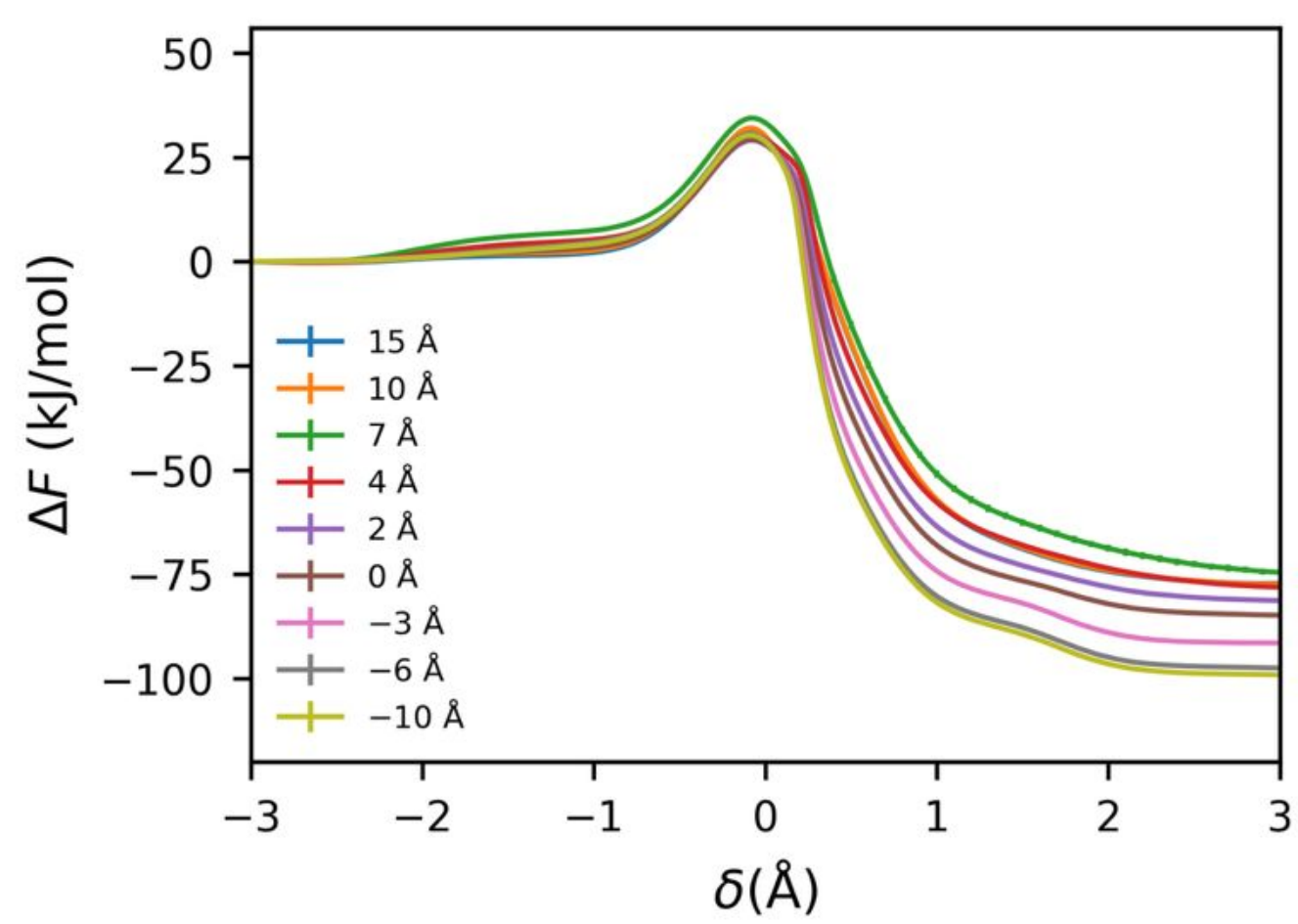

Figure S4. Reaction free energy profiles for different *z*-distances from the interface. The error bars are one standard deviation of the mean, estimated from 5 independent groups of 30 pulls, and they are too small to be visible in the plot.

Reaction free energy barriers and uncertainties in gas and condensed phases

Table S8. Reaction free energy barriers and transition–state (TS) positions for the CH_3Cl + OH reaction in the gas phase, bulk water, and at selected distances *z* from the air–water interface. One–standard–deviation uncertainties were obtained from block analysis of the SMD trajectories. Positive *z* values correspond to the gas phase side, negative to the aqueous phase.

Systems	Cases	TS position / Å	Forward barrier $\Delta F^{\ddagger}$ / kJ·mol^{-1}	Uncertainty / kJ·mol^{-1}
**Gas**	4w	−0.082	25.8479	±0.0386
**Bulk**	128w	−0.085	30.1316	±0.0530
	500w	−0.087	29.9853	±0.0460
	1000w	0.087	30.2212	±0.0551
**Interface**	$z = 15$	−0.085	31.4245	±0.1551
	$z = 10$	−0.085	31.9723	±0.2091
	$z = 7$	−0.078	34.3660	±0.0504
	$z = 4$	−0.078	30.2894	±0.0259
	$z = 2$	−0.080	29.0045	±0.0161
	$z = 0$	−0.081	29.2568	±0.0366
	$z = -3$	−0.085	30.3798	±0.0594
	$z = -6$	−0.088	30.6555	±0.0368
	$z = -10$	−0.087	30.2535	±0.0466

QM/MM simulations

For comparison, we also performed quantum mechanics/molecular mechanics (QM/MM) simulations for a spherical droplet to obtain the free energy profiles of the reaction with constant *NVT* dynamics at 300 K using umbrella sampling. The non-PBC system consists of a droplet containing 1000 water molecules described by the classical SPC/Fw water model[29] and reactant species (CH_3Cl + OH radical) described at the revPBE0+D3 level[2,30,31] with the TZVP basis set.

All the QM calculations were carried out using TeraChem.[32] The MM calculations and the dynamics were carried out using OpenMM 8.2.[33] The TeraChem protocol buffers client (TCPC)[34] interface was used for communication between TeraChem and OpenMM in the simulations.

Electrostatic embedding was used so that the QM region can be polarized by the MM region, where the MM point charges entered the Hamiltonian in the QM calculation.[35,36] The non-electrostatic part of the interactions between QM and MM atoms was handled by the LJ potential. The cross-interaction LJ parameters were obtained by applying the conventional Lorentz-Berthelot combining rules on the LJ parameters of SPC/Fw water[29] and LJ parameters from GAFF[37] for the QM atoms. No cutoff was used in the MM calculations. The temperature was maintained at 300 K using the Langevin thermostat with a frictional coefficient of 5 ps^{-1}, and the time step was 0.5 fs. At 300 K and equilibrium, the droplet radius was around 19.3 Å. The center of mass (COM) of the droplet was restrained at the origin with a force constant of 10 kJ/mol/$Å^2$ using a harmonic potential. A harmonic wall potential with a force constant of 10 kJ/mol/$Å^2$ was set at 30 Å away from the COM of the droplet to prevent evaporated water from escaping. The wall potential acted on the water oxygen atoms only when they went beyond 30 Å away from the COM of the droplet. To sample the reaction at different places, the carbon atom of the CH_3Cl was restrained at 0, 13.3, 16.3, and 19.3 Å away from the COM of the droplet with a force constant of 10 kJ/mol/$Å^2$. These four distances from the droplet COM correspond to four places in the system: the droplet center, 6 Å below the interface, 3 Å below the interface, and at the interface. For each place, a set of bias center and bias spring constant values was determined for the US windows, so that the normalized biased distribution of adjacent windows crossed at roughly 0.8 standard deviation, and the mean of the CV of each window was within 0.1 Å from the window bias center. At least 54 ps of production data were collected for each window to reconstruct the free energy profiles using WHAM.[38]

Because any one of the hydrogen atoms in the CH_3Cl can be abstracted by the OH radical in the QM/MM simulation, a harmonic wall potential was used to keep two of the hydrogen atoms away from the oxygen of the OH radical. The wall potential with a force constant of 10 kJ/mol/$Å^2$ acted only when the distance between the hydrogen and oxygen atoms was less than 1.3 Å.

The calculated free energy profiles are shown in Figure S5. From the results, we find that the reaction occurring just at the interface exhibits the highest forward barrier in free energy, around 30 kJ/mol, which perfectly matches the barrier obtained from the EVB results. A slight decrease

in the forward barrier was observed as the reaction location shifted toward the inner droplet, reaching a value of 26 kJ/mol at the droplet center. The trend is not reflected in the EVB results, which showed no measurable change in the barrier when the reaction location moved from the interface to the inner bulk water.

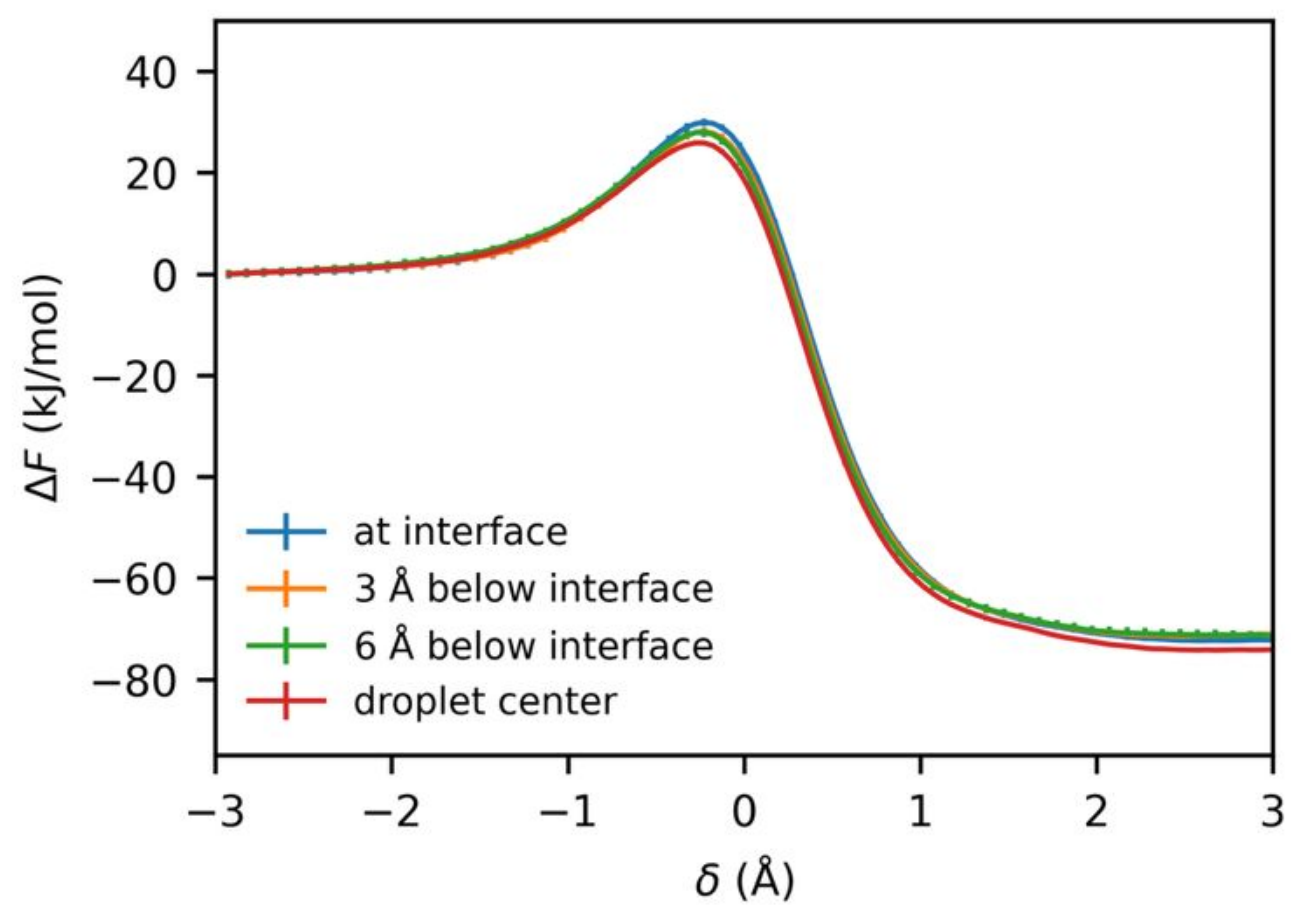

Figure S5. Free energy profiles from QM/MM umbrella sampling simulations for the reaction happening at four different locations in a 1000-water droplet system. Error bars are one standard deviation of the mean obtained from 5 independent US attempts.

Near monolayer coverage of 50 CH_3Cl molecules on a water droplet

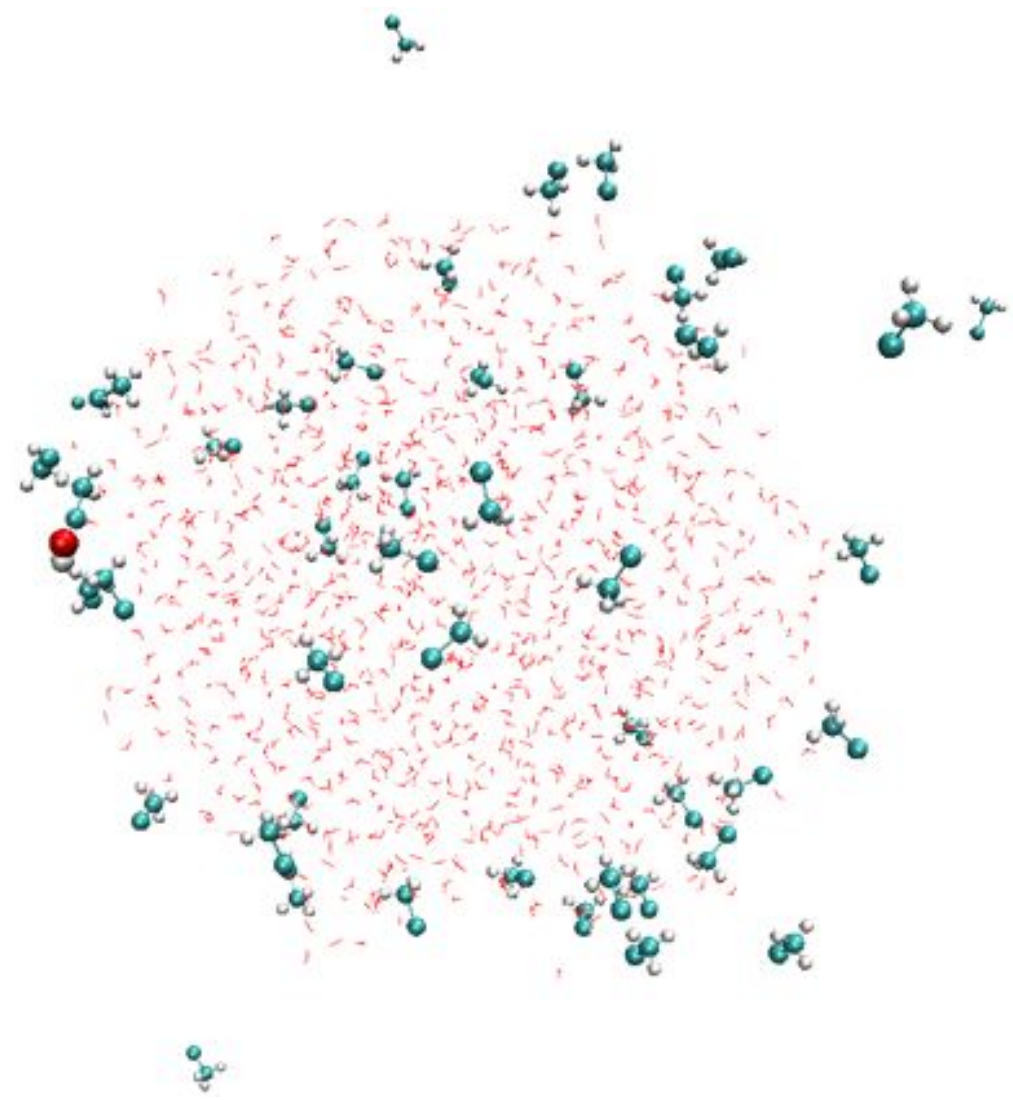

Figure S6. A snapshot showing the additional 50 CH_3Cl molecules form a sub-monolayer to near-monolayer coverage on a 1000-water droplet of radius ~19.3 Å, corresponding to an approximate areal density of 1 molecule per nm^2.

Thermodynamic limit of solvation free energies

Li et al. previously reported that the free energy of solvation ΔF_{solv} depends linearly on the inverse of the number of water molecules $1/N_w$ when N_w is sufficiently large.[39] As shown in Figure S7, similar linear scaling has been observed over the range $1/2000 < 1/N_w < 1/500$ for solvating a single CH_3Cl molecule in a water droplet. Therefore, the free energy of solvation in the thermodynamic limit can be extrapolated at the y-intercept, where $1/N_w \rightarrow 0$. For CH_3Cl solvated into a water droplet, ΔF_{solv} is about 0.19 kcal/mol in the thermodynamic limit. This result accords with the reported experimental value of –0.55 kcal/mol[40] and the recently reported computational reference values between –1.482 and 0.884 kcal/mol.[41] Similar to the results of CH_3Cl, a linear relationship between $1/N_w$ and ΔF_{solv} for OH radical also exists and is shown in Figure S8. The thermodynamic limit for $1/N_w \rightarrow 0$ is about –4.9 kcal/mol.

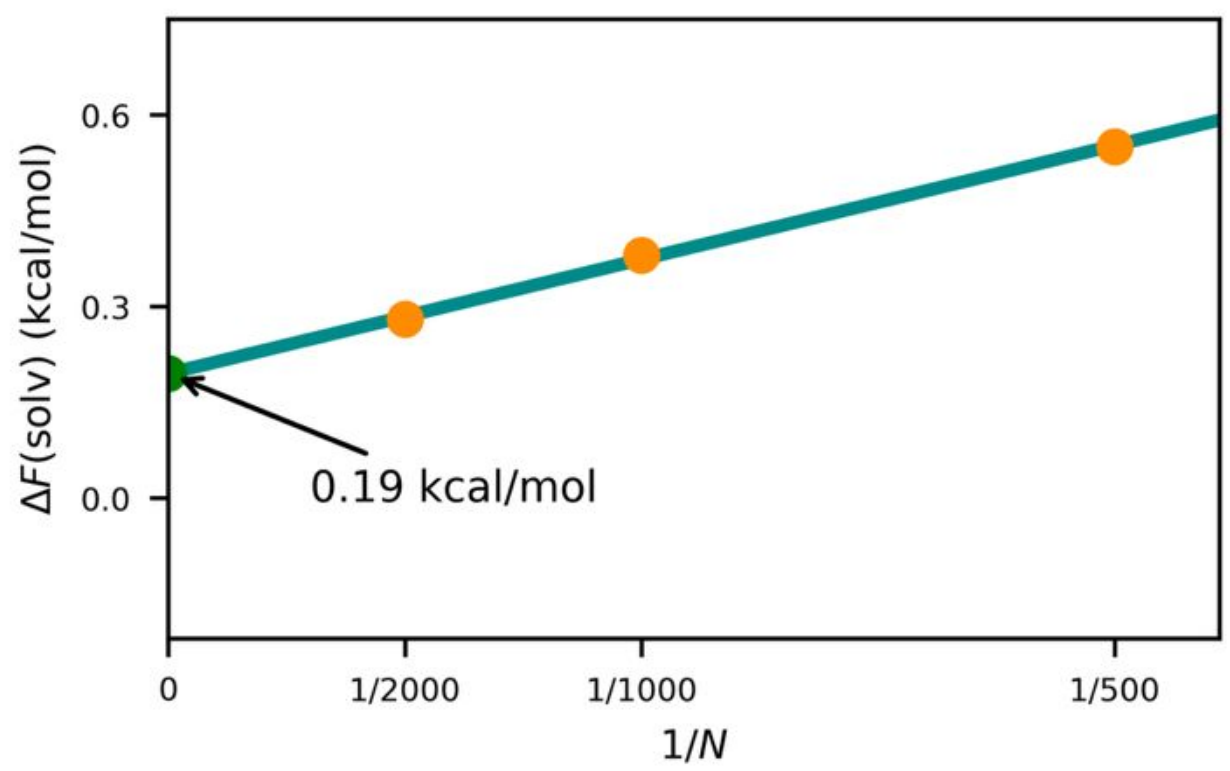

Figure S7. Calculated solvation free energies of CH_3Cl (extracted from Figure 9 in the main text) as a function of the reciprocal of the number of water molecules $1/N$ in the water droplet. These results were extrapolated to the $1/N \rightarrow 0$ limit, corresponding to an infinitely large system.

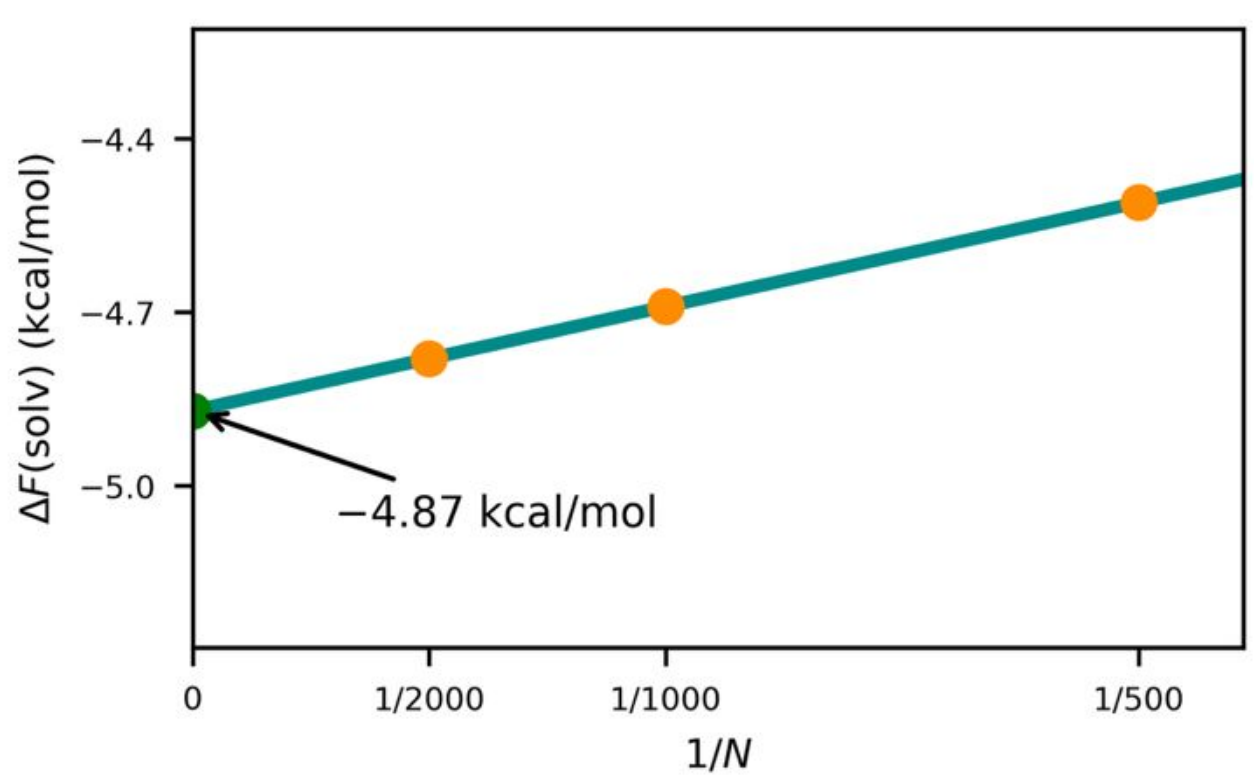

Figure S8. Calculated solvation free energies of OH (extracted from Figure 9 in the main text) as a function of the reciprocal of the number of water molecules $1/N$ in the water droplet. These results were extrapolated to the $1/N \rightarrow 0$ limit, corresponding to an infinitely large system.

Free energy study of CH_3Cl and OH sorption by water slabs

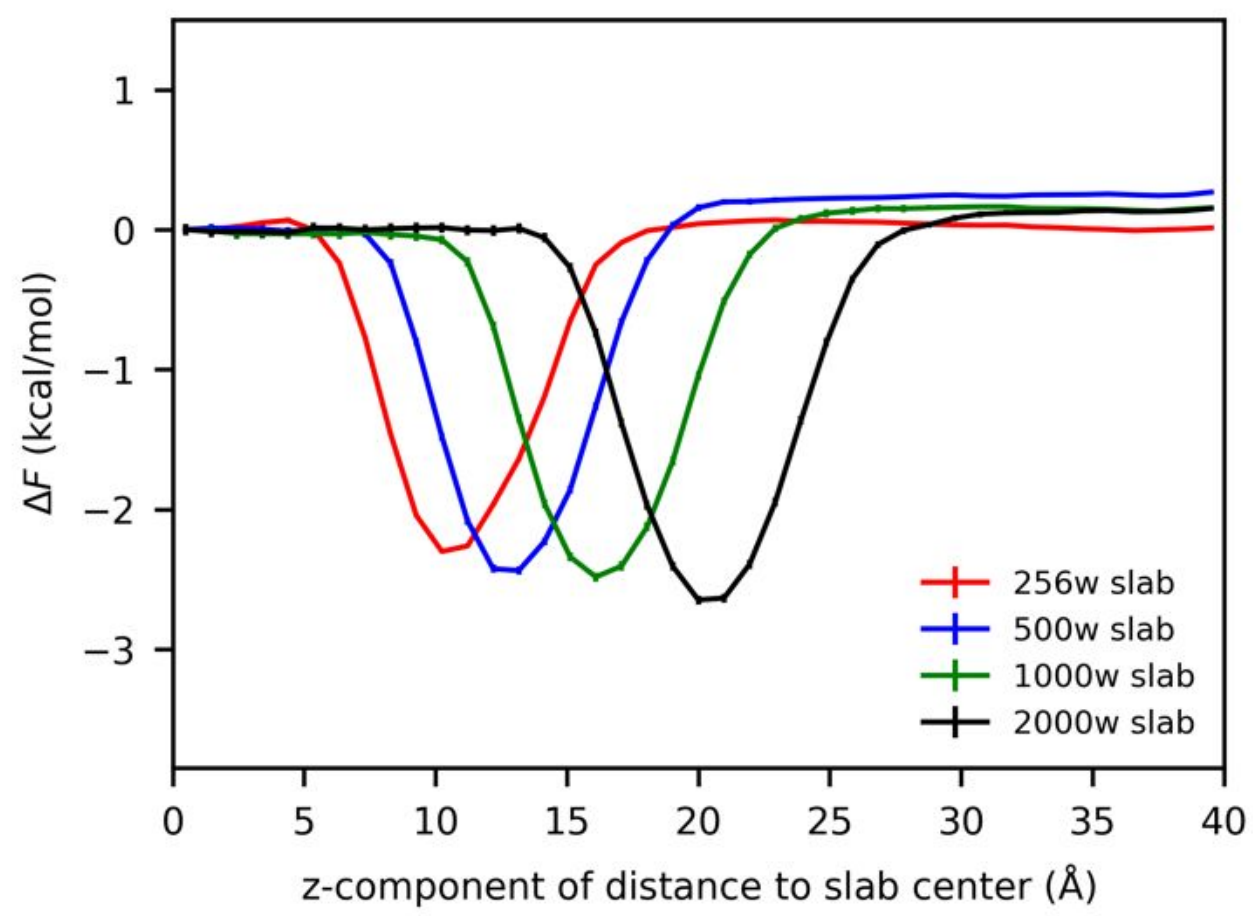

Figure S9. Free energy profiles for CH_3Cl as a function of the *z* distance between the COM of the gas molecule and a slab of 256 (red), 500 (blue), 1000 (green), or 2000 (black) water molecules. The error bars were obtained by bootstrapping. As indicated by the dip in the free energy profile at the droplet interface, there is an apparent propensity of the CH_3Cl at the interface, as we discussed in the main text for the droplet geometry. CH_3Cl can accumulate at the interface, resulting in an excess concentration compared to the gas or aqueous phases.

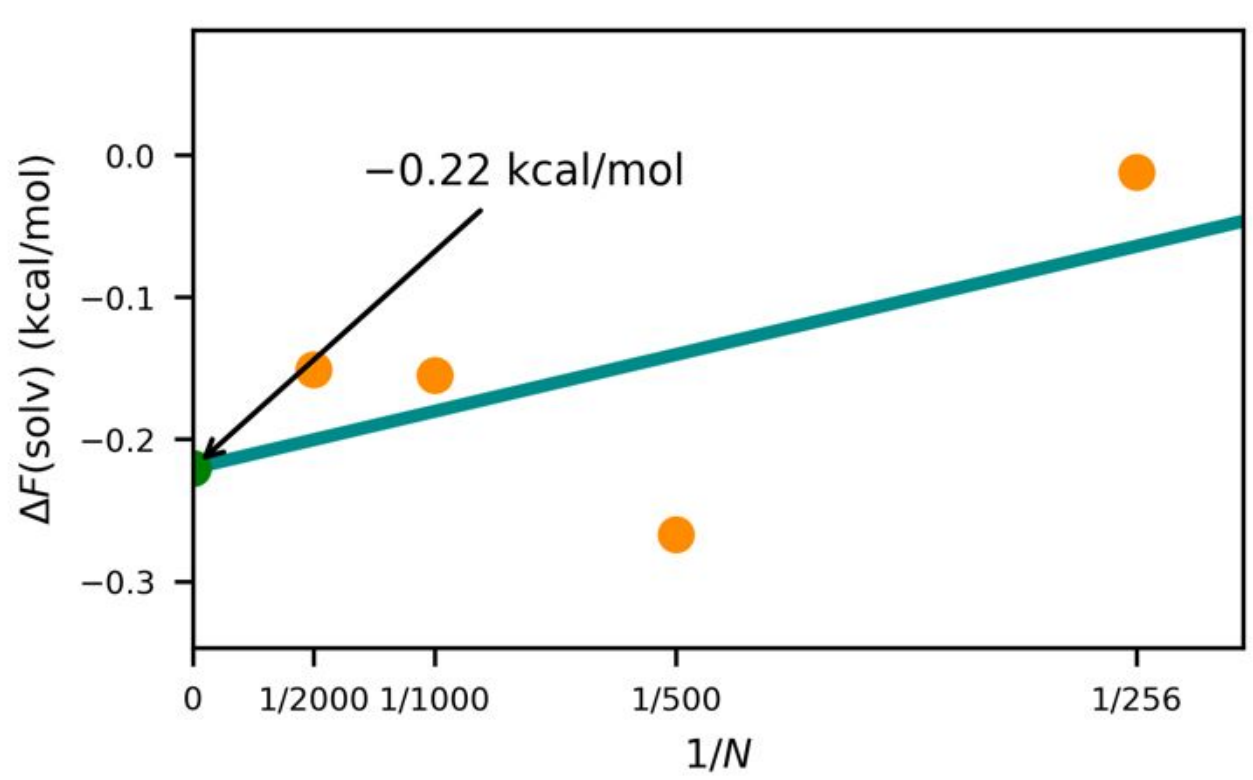

Figure S10. Calculated solvation free energies of CH_3Cl (extracted from Figure S9) as a function of the reciprocal of the number of water molecules $1/N$ in the water slab. These results were extrapolated to the $1/N \rightarrow 0$ limit, corresponding to an infinitely large system.

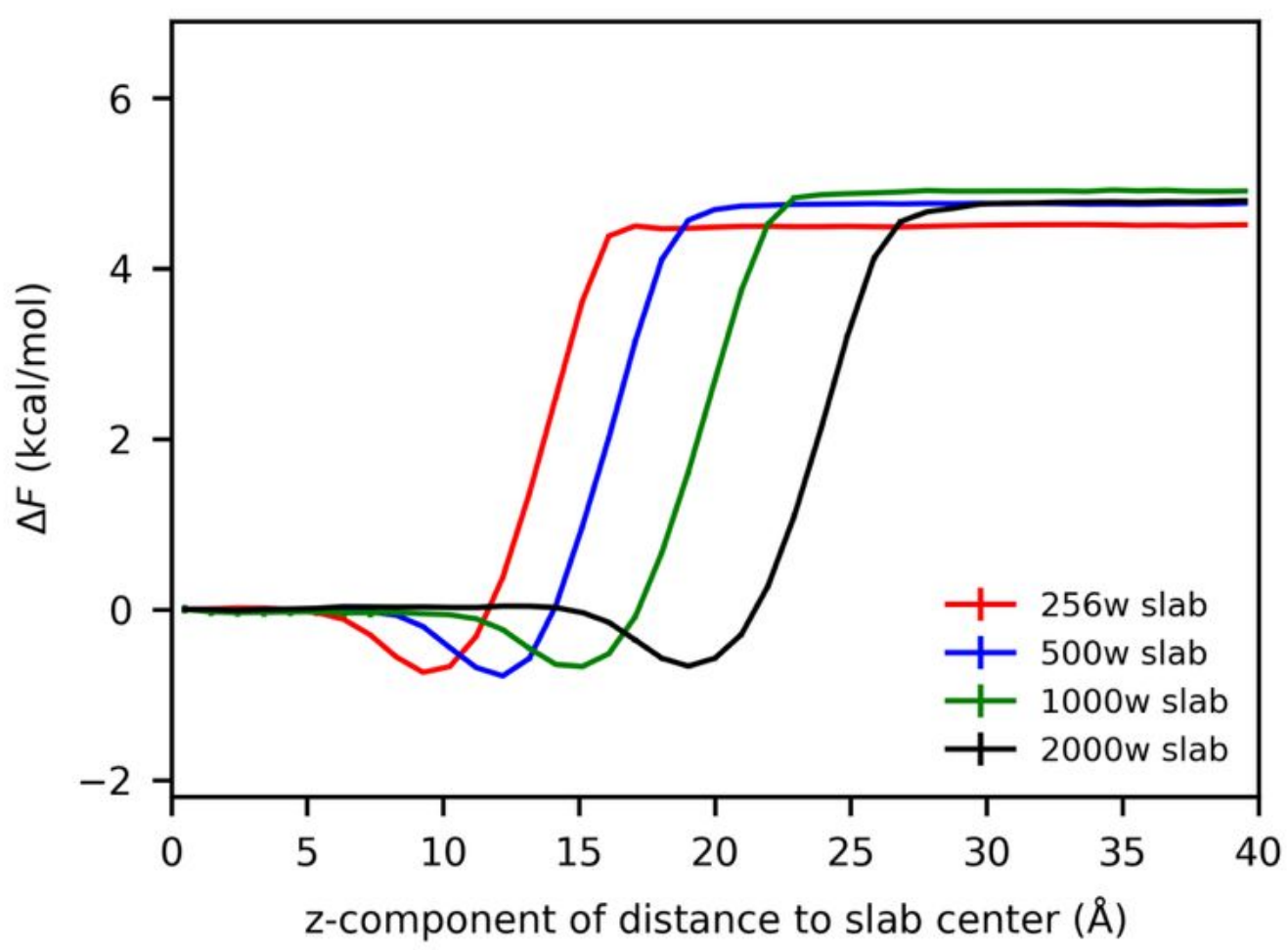

Figure S11. Free energy profiles for OH as a function of the z distance between the COM of the gas molecule and a slab of 256 (red), 500 (blue), 1000 (green), or 2000 (black) water molecules. The error bars were obtained by bootstrapping. As indicated by the small dip in the free energy profile at the droplet interface, there is a small propensity of the OH at the interface, as we discussed in the main text for the droplet geometry. The OH prefers to stay near the interface and within the droplet, rather than remaining in the gas phase.

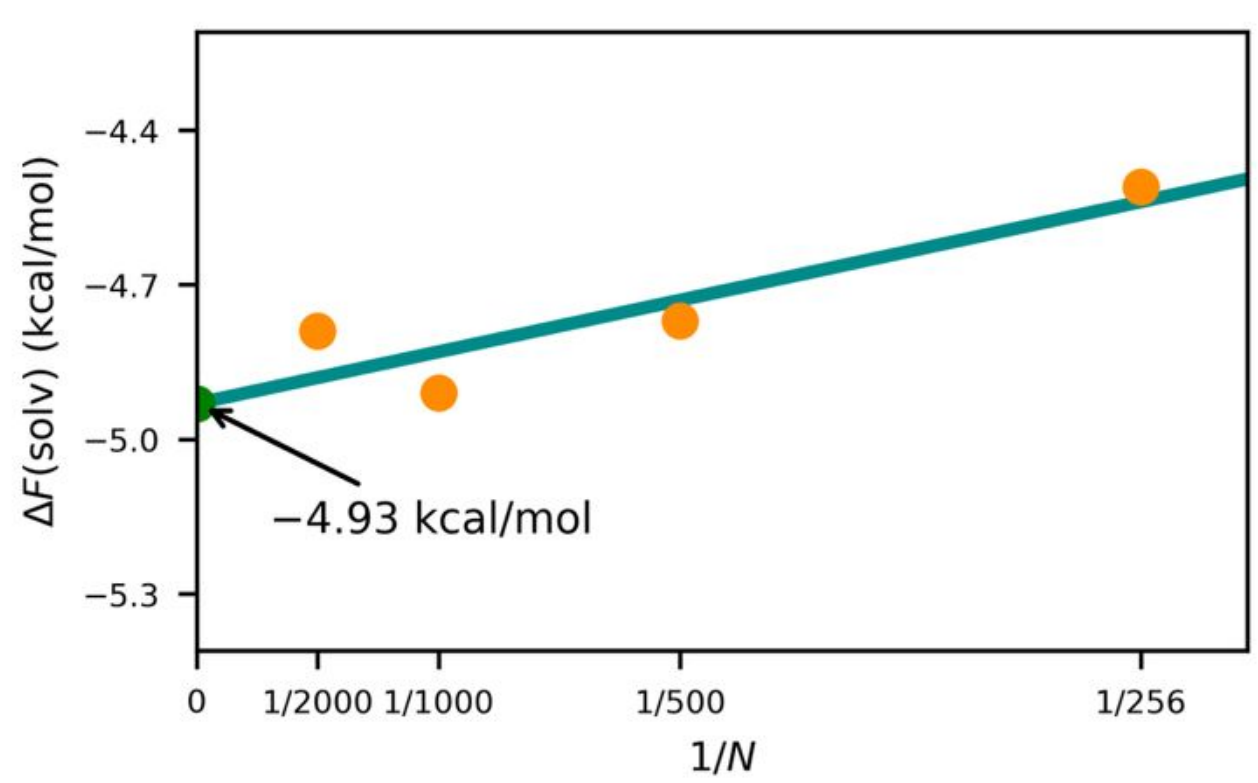

Figure S12. Calculated solvation free energies of OH (extracted from Figure S11) as a function of the reciprocal of the number of water molecules $1/N$ in the water slab. These results were extrapolated to the $1/N \rightarrow 0$ limit, corresponding to an infinitely large system.

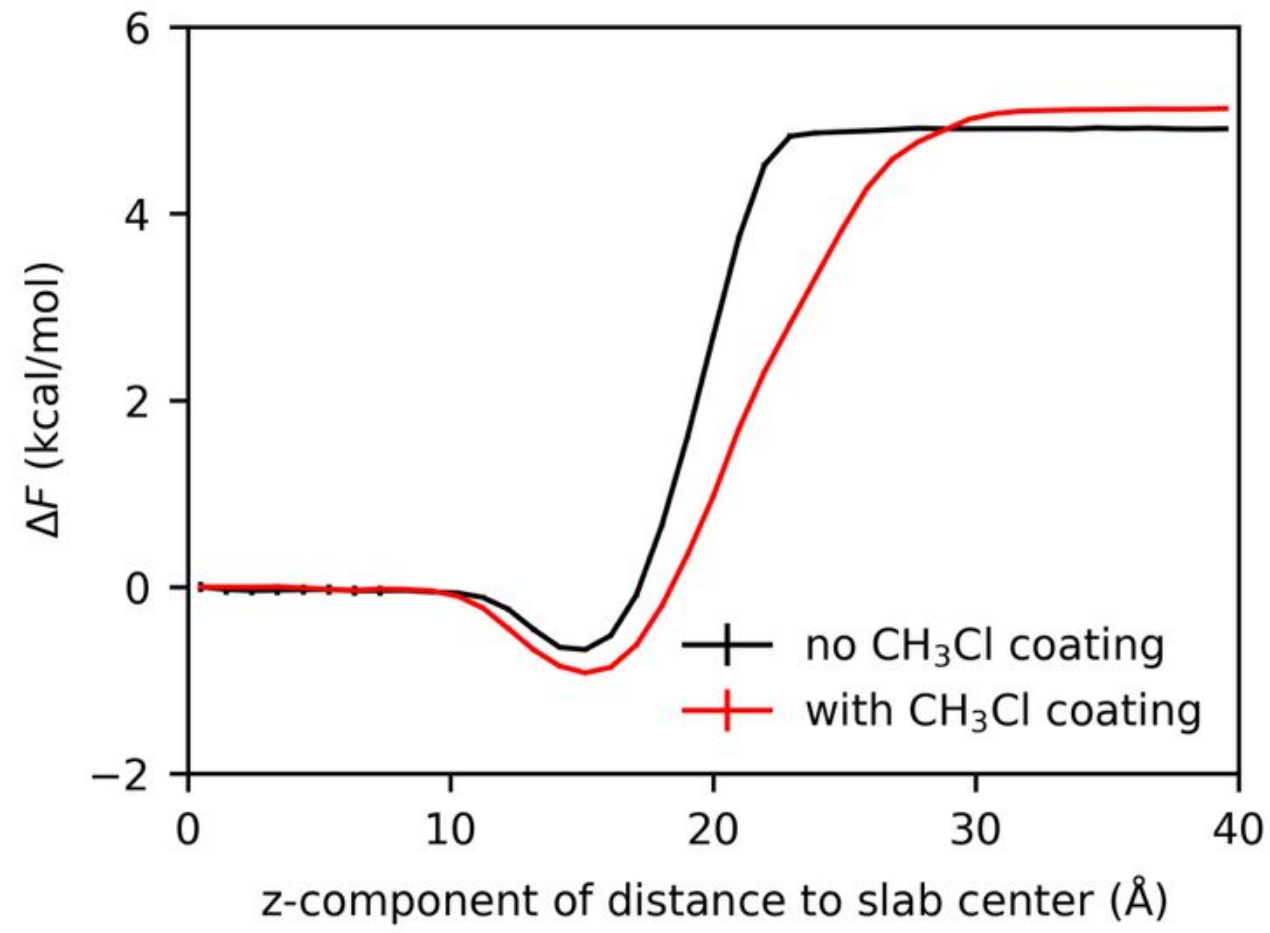

Figure S13. Free energy profiles of solvating one OH radical into a 1000-water slab, with and without 50 additional CH_3Cl molecules forming a coating layer. The error bars were obtained by bootstrapping. Similar to the droplet geometry results presented in Figure 10 in the main text, for the water slab with 1000 water molecules, there is a slightly greater tendency for the OH radical to accumulate at the interface when additional CH_3Cl molecules form an organic layer at the interface.

Error propagation from free energy to rate constant

To quantify how the statistical uncertainty in $\Delta F^{\ddagger}$ affects the rate constants, we propagated the errors from the free energy calculations through the Eyring equation

$$k = \frac{\kappa k_{\mathrm{B}} T}{h} \exp\left(-\frac{\Delta F^{\ddagger}}{RT}\right)$$

Write the rate as

$$\ln k = \ln\left(\frac{\kappa k_{\mathrm{B}} T}{h}\right) - \frac{\Delta F^{\ddagger}}{RT}$$

The uncertainty in $\ln k$ arising from an uncertainty $\sigma_{\Delta F^{\ddagger}}$ in $\Delta F^{\ddagger}$ is

$$\sigma_{\ln k} = \frac{\sigma_{\Delta F^{\ddagger}}}{RT}$$

The corresponding multiplicative uncertainty ("error factor") in the rate constant is therefore

$$f_k = \exp\left(\frac{\sigma_{\Delta F^{\ddagger}}}{RT}\right)$$

so that the rate constant lies within a factor f_k of its mean value. For example, an uncertainty of 1~kJ/mol in $\Delta F^{\ddagger}$ at T = 300 K (where $RT \approx 2.48$ kJ/mol) corresponds to $f_k \approx \exp\left(\frac{1}{2.48}\right) \approx 1.5$.

References

(1) Zhao, Y.; Truhlar, D. G. The M06 Suite of Density Functionals for Main Group Thermochemistry, Thermochemical Kinetics, Noncovalent Interactions, Excited States, and Transition Elements: Two New Functionals and Systematic Testing of Four M06-Class Functionals and 12 Other Functionals. *Theor. Chem. Acc.* **2008**, *120* (1–3), 215–241. https://doi.org/10.1007/s00214-007-0310-x.

(2) Grimme, S.; Antony, J.; Ehrlich, S.; Krieg, H. A Consistent and Accurate *Ab Initio* Parametrization of Density Functional Dispersion Correction (DFT-D) for the 94 Elements H-Pu. *J. Chem. Phys.* **2010**, *132* (15), 154104. https://doi.org/10.1063/1.3382344.

(3) Weigend, F.; Ahlrichs, R. Balanced Basis Sets of Split Valence, Triple Zeta Valence and Quadruple Zeta Valence Quality for H to Rn: Design and Assessment of Accuracy. *Phys. Chem. Chem. Phys.* **2005**, *7* (18), 3297. https://doi.org/10.1039/b508541a.

(4) Zheng, J.; Xu, X.; Truhlar, D. G. Minimally Augmented Karlsruhe Basis Sets. *Theor. Chem. Acc.* **2011**, *128* (3), 295–305. https://doi.org/10.1007/s00214-010-0846-z.

(5) Riplinger, C.; Neese, F. An Efficient and near Linear Scaling Pair Natural Orbital Based Local Coupled Cluster Method. *J. Chem. Phys.* **2013**, *138* (3), 034106. https://doi.org/10.1063/1.4773581.

(6) Riplinger, C.; Sandhoefer, B.; Hansen, A.; Neese, F. Natural Triple Excitations in Local Coupled Cluster Calculations with Pair Natural Orbitals. *J. Chem. Phys.* **2013**, *139* (13), 134101. https://doi.org/10.1063/1.4821834.

(7) Riplinger, C.; Pinski, P.; Becker, U.; Valeev, E. F.; Neese, F. Sparse Maps—A Systematic Infrastructure for Reduced-Scaling Electronic Structure Methods. II. Linear Scaling Domain Based Pair Natural Orbital Coupled Cluster Theory. *J. Chem. Phys.* **2016**, *144* (2), 024109. https://doi.org/10.1063/1.4939030.

(8) Saitow, M.; Becker, U.; Riplinger, C.; Valeev, E. F.; Neese, F. A New Near-Linear Scaling, Efficient and Accurate, Open-Shell Domain-Based Local Pair Natural Orbital Coupled Cluster Singles and Doubles Theory. *J. Chem. Phys.* **2017**, *146* (16), 164105. https://doi.org/10.1063/1.4981521.

(9) Guo, Y.; Riplinger, C.; Becker, U.; Liakos, D. G.; Minenkov, Y.; Cavallo, L.; Neese, F. Communication: An Improved Linear Scaling Perturbative Triples Correction for the Domain Based Local Pair-Natural Orbital Based Singles and Doubles Coupled Cluster Method [DLPNO-CCSD(T)]. *J. Chem. Phys.* **2018**, *148* (1), 011101. https://doi.org/10.1063/1.5011798.

(10) Kendall, R. A.; Dunning, T. H.; Harrison, R. J. Electron Affinities of the First-Row Atoms Revisited. Systematic Basis Sets and Wave Functions. *J. Chem. Phys.* **1992**, *96* (9), 6796–6806. https://doi.org/10.1063/1.462569.

(11) Becke, A. D. Density-Functional Exchange-Energy Approximation with Correct Asymptotic Behavior. *Phys. Rev. A* **1988**, *38* (6), 3098–3100. https://doi.org/10.1103/PhysRevA.38.3098.

(12) Lee, C.; Yang, W.; Parr, R. G. Development of the Colle-Salvetti Correlation-Energy Formula into a Functional of the Electron Density. *Phys. Rev. B* **1988**, *37* (2), 785–789. https://doi.org/10.1103/PhysRevB.37.785.

(13) Miehlich, B.; Savin, A.; Stoll, H.; Preuss, H. Results Obtained with the Correlation Energy Density Functionals of Becke and Lee, Yang and Parr. *Chem. Phys. Lett.* **1989**, *157* (3), 200–206. https://doi.org/10.1016/0009-2614(89)87234-3.

(14) Becke, A. D. Density-Functional Thermochemistry. III. The Role of Exact Exchange. *J. Chem. Phys.* **1993**, *98* (7), 5648–5652. https://doi.org/10.1063/1.464913.

(15) Stephens, P. J.; Devlin, F. J.; Chabalowski, C. F.; Frisch, M. J. Ab Initio Calculation of Vibrational Absorption and Circular Dichroism Spectra Using Density Functional Force Fields. *J. Phys. Chem.* **1994**, *98* (45), 11623–11627. https://doi.org/10.1021/j100096a001.

(16) Mardirossian, N.; Head-Gordon, M. *ω* B97M-V: A Combinatorially Optimized, Range-Separated Hybrid, Meta-GGA Density Functional with VV10 Nonlocal Correlation. *J. Chem. Phys.* **2016**, *144* (21), 214110. https://doi.org/10.1063/1.4952647.

(17) Lu, T.; Chen, Q. Interaction Region Indicator: A Simple Real Space Function Clearly Revealing Both Chemical Bonds and Weak Interactions. *Chemistry–Methods* **2021**, *1* (5), 231–239. https://doi.org/10.1002/cmtd.202100007.

(18) Lu, T.; Chen, F. Multiwfn: A Multifunctional Wavefunction Analyzer. *J. Comput. Chem.* **2012**, *33* (5), 580–592. https://doi.org/10.1002/jcc.22885.

(19) Lu, T. A Comprehensive Electron Wavefunction Analysis Toolbox for Chemists, Multiwfn. *J. Chem. Phys.* **2024**, *161* (8), 082503. https://doi.org/10.1063/5.0216272.

(20) Thole, B. T. Molecular Polarizabilities Calculated with a Modified Dipole Interaction. *Chem. Phys.* **1981**, *59* (3), 341–350.

(21) Yu, H.; Whitfield, T. W.; Harder, E.; Lamoureux, G.; Vorobyov, I.; Anisimov, V. M.; MacKerell Jr, A. D.; Roux, B. Simulating Monovalent and Divalent Ions in Aqueous Solution Using a Drude Polarizable Force Field. *J. Chem. Theory Comput.* **2010**, *6* (3), 774–786.

(22) Lamoureux, G.; MacKerell, A. D.; Roux, B. A Simple Polarizable Model of Water Based on Classical Drude Oscillators. *J. Chem. Phys.* **2003**, *119* (10), 5185–5197. https://doi.org/10.1063/1.1598191.

(23) Lamoureux, G.; Roux, B. Modeling Induced Polarization with Classical Drude Oscillators: Theory and Molecular Dynamics Simulation Algorithm. *J. Chem. Phys.* **2003**, *119* (6), 3025–3039. https://doi.org/10.1063/1.1589749.

(24) Lamoureux, G.; Harder, E.; Vorobyov, I. V.; Roux, B.; MacKerell, A. D. A Polarizable Model of Water for Molecular Dynamics Simulations of Biomolecules. *Chem. Phys. Lett.* **2006**, *418* (1–3), 245–249. https://doi.org/10.1016/j.cplett.2005.10.135.

(25) Wang, X.; Tse, Y.-L. S. Flexible Polarizable Water Model Parameterized via Gaussian Process Regression. *J. Chem. Theory Comput.* **2022**, *18* (12), 7155–7165. https://doi.org/10.1021/acs.jctc.2c00529.

(26) Boys, S. F.; Bernardi, F. The Calculation of Small Molecular Interactions by the Differences of Separate Total Energies. Some Procedures with Reduced Errors. *Mol. Phys.* **1970**, *19* (4), 553–566. https://doi.org/10.1080/00268977000101561.

(27) Simon, S.; Duran, M.; Dannenberg, J. J. How Does Basis Set Superposition Error Change the Potential Surfaces for Hydrogen-Bonded Dimers? *J. Chem. Phys.* **1996**, *105* (24), 11024–11031. https://doi.org/10.1063/1.472902.

(28) Lu, T.; Chen, Q. Shermo: A General Code for Calculating Molecular Thermochemistry Properties. *Comput. Theor. Chem.* **2021**, *1200*, 113249. https://doi.org/10.1016/j.comptc.2021.113249.
(29) Wu, Y.; Tepper, H. L.; Voth, G. A. Flexible Simple Point-Charge Water Model with Improved Liquid-State Properties. *J. Chem. Phys.* **2006**, *124* (2), 024503. https://doi.org/10.1063/1.2136877.
(30) Zhang, Y.; Yang, W. Comment on "Generalized Gradient Approximation Made Simple." *Phys. Rev. Lett.* **1998**, *80* (4), 890–890. https://doi.org/10.1103/PhysRevLett.80.890.
(31) Adamo, C.; Barone, V. Toward Reliable Density Functional Methods without Adjustable Parameters: The PBE0 Model. *J. Chem. Phys.* **1999**, *110* (13), 6158–6170. https://doi.org/10.1063/1.478522.
(32) Ufimtsev, I. S.; Martinez, T. J. Quantum Chemistry on Graphical Processing Units. 3. Analytical Energy Gradients, Geometry Optimization, and First Principles Molecular Dynamics. *J. Chem. Theory Comput.* **2009**, *5* (10), 2619–2628. https://doi.org/10.1021/ct9003004.
(33) Eastman, P.; Galvelis, R.; Peláez, R. P.; Abreu, C. R. A.; Farr, S. E.; Gallicchio, E.; Gorenko, A.; Henry, M. M.; Hu, F.; Huang, J.; Krämer, A.; Michel, J.; Mitchell, J. A.; Pande, V. S.; Rodrigues, J. P.; Rodriguez-Guerra, J.; Simmonett, A. C.; Singh, S.; Swails, J.; Turner, P.; Wang, Y.; Zhang, I.; Chodera, J. D.; De Fabritiis, G.; Markland, T. E. OpenMM 8: Molecular Dynamics Simulation with Machine Learning Potentials. *J. Phys. Chem. B* **2024**, *128* (1), 109–116. https://doi.org/10.1021/acs.jpcb.3c06662.
(34) Cruzeiro, V. W. D.; Wang, Y.; Pieri, E.; Hohenstein, E. G.; Martínez, T. J. TeraChem Protocol Buffers (TCPB): Accelerating QM and QM/MM Simulations with a Client–Server Model. *J. Chem. Phys.* **2023**, *158* (4), 044801. https://doi.org/10.1063/5.0130886.
(35) Lin, H. *QM/MM Methods*; ACS In Focus; American Chemical Society: Washington, DC, USA, 2024. https://doi.org/10.1021/acsinfocus.7e7030.
(36) Senn, H. M.; Thiel, W. QM/MM Methods for Biomolecular Systems. *Angew. Chem. Int. Ed.* **2009**, *48* (7), 1198–1229. https://doi.org/10.1002/anie.200802019.
(37) Wang, J.; Wolf, R. M.; Caldwell, J. W.; Kollman, P. A.; Case, D. A. Development and Testing of a General Amber Force Field. *J. Comput. Chem.* **2004**, *25* (9), 1157–1174. https://doi.org/10.1002/jcc.20035.
(38) Kumar, S.; Rosenberg, J. M.; Bouzida, D.; Swendsen, R. H.; Kollman, P. A. The Weighted Histogram Analysis Method for Free-Energy Calculations on Biomolecules. I. The Method. *J. Comput. Chem.* **1992**, *13* (8), 1011–1021. https://doi.org/10.1002/jcc.540130812.
(39) Li, W.; Pak, C. Y.; Tse, Y.-L. S. Free Energy Study of H2O, N2O5, SO2, and O3 Gas Sorption by Water Droplets/Slabs. *J. Chem. Phys.* **2018**, *148* (16), 164706. https://doi.org/10.1063/1.5022389.
(40) Rizzo, R. C.; Aynechi, T.; Case, D. A.; Kuntz, I. D. Estimation of Absolute Free Energies of Hydration Using Continuum Methods: Accuracy of Partial Charge Models and Optimization of Nonpolar Contributions. *J. Chem. Theory Comput.* **2006**, *2* (1), 128–139. https://doi.org/10.1021/ct050097l.
(41) Bensberg, M.; Türtscher, P. L.; Unsleber, J. P.; Reiher, M.; Neugebauer, J. Solvation Free Energies in Subsystem Density Functional Theory. *J. Chem. Theory Comput.* **2022**, *18* (2), 723–740. https://doi.org/10.1021/acs.jctc.1c00864.
